# A Randomized, Placebo Controlled, Double Masked Phase IB Study Evaluating the Safety and Antiviral Activity of Aprepitant, a Neurokinin-1 Receptor Antagonist in HIV-1 Infected Adults

**DOI:** 10.1371/journal.pone.0024180

**Published:** 2011-09-08

**Authors:** Pablo Tebas, Florin Tuluc, Jeffrey S. Barrett, Wayne Wagner, Deborah Kim, Huaquing Zhao, René Gonin, James Korelitz, Steven D. Douglas

**Affiliations:** 1 Division of Infectious Diseases, University of Pennsylvania, Philadelphia, Pennsylvania, United States of America; 2 The Children's Hospital of Philadelphia, Philadelphia, Pennsylvania, United States of America; 3 Westat, Rockville, Maryland, United States of America; Mayo Clinic, United States of America

## Abstract

**Background:**

Neurokinin-1 receptor (NK1R) antagonists have anti-HIV activity in monocyte-derived macrophages, decrease CCR5 expression and improve natural killer cell function *ex vivo*. Aprepitant is a NK1R antagonist approved by FDA as an antiemetic.

**Methods:**

We conducted a phase IB randomized, placebo controlled, double masked study to evaluate the safety, antiviral activity, pharmacokinetics and immune-modulatory effects of aprepitant in HIV-infected adults not receiving antiretroviral therapy, with CD4+ cell count ≥350 cells/mm^3^ and plasma viral load ≥2,000 copies/ml. Subjects were stratified by viral load (< vs. ≥20,000 copies/ml) and randomized within each stratum to receive aprepitant at 125 mg QD(Low), or 250 mg QD(High), or placebo(PL) for 14 days, and followed for 42 days.

**Results:**

Thirty subjects were randomized and 27 completed treatment (9, 8, 10 subjects in 125 (Low), 250 (High), and PL groups). 63% were male; 37% white; mean (SD) age 43 (9.3) years. Geometric mean baseline viral load (copies/ml) for Low, High, and PL was 15,709, 33,013, and 19,450, respectively. Mean (95%CI) change in log10 viral load at day 14 for Low, High, and PL was −0.02(−0.24,+0.20), −0.05(−0.21,+0.10), and +0.04(−0.08,+0.16), respectively. The number of subjects with AEs was 4(44.4%), 5(62.5%), and 1(10%) for Low, High, and PL. No Grade 4 AEs occurred.

**Conclusions:**

Adverse events of aprepitant were more common in the treated groups. At the dose used in this two-week phase IB study, aprepitant showed biological activity, but no significant antiviral activity.

**Trial Registration:**

ClinicalTrials.gov NCT00428519

## Introduction

The current paradigm of HIV treatment is the continuous use of multiple antiretroviral drugs that target several steps of the life cycle of the HIV virus. This strategy has been associated with substantial decreases in the morbidity and mortality associated with HIV infection [1]. Despite the success of antiretroviral therapy, treatment failure occurs in many patients. In spite of the recent approval of new classes of drugs for the treatment of HIV infection [2], [3], [4], it is predictable that resistance will ultimately develop to these new agents too, and that new antivirals will be required.

A continued challenge in anti-HIV drug development is to make available antiretroviral agents with new target and mechanisms of action and activity against drug-resistant virus. Alternative paradigms for the treatment of HIV infection also need to be explored. Recent studies have demonstrated the key role that immune activation plays in the pathogenesis of HIV infection and disease progression [5], [6], [7], [8], [9]. Patients with HIV infection, even if successfully treated also have a high frequency of neurocognitive impairment that it is associated with this residual chronic inflammation [10], [11].

The use of pharmacological agents and immunomodulators that target this residual inflammation acting at the cellular level, rather than at the HIV virus level might be useful in preventing inflammatory and neurocognitive events associated with HIV infection [12], [13]. The use of neurokinin-1 receptors (substance P preferring) antagonists in the management of HIV infection can serve both purposes, as these compounds may have both antiviral and immunomodulatory effects. Furthermore, these agents cross the blood-brain barrier and have anti-depressive behavior activity.

Substance P (SP), a member of the tachykinin family [14]; plays a central modulator role in neuroimmunoregulation, in particular, the immune functions of mononuclear phagocytes, but also other immune cells [15]. SP and its receptor, NK1R, may be important and have modulatory effects in HIV-infected individuals. Our group has demonstrated elevated plasma levels of SP in HIV-positive men [16] in comparison with high-risk HIV-negative men, and also in HIV positive women [17]. In a series of in vitro experiments we demonstrated that SP-antagonists (CP-96,345) can inhibit HIV replication in macrophages in a concentration dependent manner in vitro [18]. We also have shown that this inhibition of HIV viral replication is mediated to the binding of CP-96,345 to the NK1R and may be mediated by a decreased expression of the HIV coreceptor CCR5 [18]. In addition, we have demonstrated synergy of aprepitant with other antiviral drugs [19] in *ex vivo* studied.

Preliminary data suggest that aprepitant, the only approved SP antagonist has anti-HIV activity similar to CP-96,345 in the same ex vivo system [20]. We studied the anti-HIV-l activity of aprepitant against multiple macrophage tropic (R5) and T-cell tropic (×4) HIV-1 isolates grown in cell culture. Aprepitant had the highest anti-HIV-1 activity of the NK1R antagonists examined and was equally active against all major HIV-1 subtypes. Aprepitant acted synergistically with protease inhibitors (ritonavir and saquinavir), but not with nucleoside reverse transcriptase, non-nucleoside reverse transcriptase, or viral entry inhibitors [19].

The purpose of this clinical trial was to determine the in vivo safety and antiviral activity of aprepitant by comparing the change in HIV RNA viral load after 2 weeks of aprepitant monotherapy in patients with HIV infection not receiving any other antiretroviral treatment. The study was designed as a two-week trial as mandated by the FDA in order to avoid emergence of resistance or changes in tropism if aprepitant had significant antiviral activity.

## Methods

The protocol for this trial and supporting CONSORT checklist are available as supporting information; see Checklist S1 and Protocol S1.

### Study design and study procedures

This was a phase IB randomized, placebo controlled, double masked study to evaluate the safety, antiviral activity, pharmacokinetics and immune modulatory effects of aprepitant in HIV infected adults not receiving antiretroviral therapy, with CD4+ cell count ≥350 cells/mm^3^ and plasma viral load ≥2,000 copies/ml. Thirty patients with HIV-1 infection, not receiving antiretroviral therapy were stratified by viral load (< vs. ≥20,000 copies/ml) and randomized within each stratum to receive aprepitant 125 mg QD(Low), 250 mg QD(High), or placebo (PL) for 14 days, and followed for 42 days. The drug was masked by over encapsulation. The investigators were blinded to the study assignment of the patients.

At the screening visit, previous antiretroviral treatment (if any) was assessed, safety laboratory tests were conducted, and all patients underwent testing for HIV-1 co-receptor tropism with the use of a validated phenotypic tropism assay (Trofile, Monogram Biosciences). Patients were also tested for plasma levels of HIV-1 RNA (Amplicor HIV-1 Monitor v1.5, Roche Diagnostics). Participants were then randomized and evaluated at day 0, 3, 7, 10, 14, while they were receiving aprepitant or placebo, and at day 42, 4 weeks after discontinuing study medication. Additionally, and 8 hour pharmacokinetic assessment was done after the first dose and at day 14.

### SP levels

#### SP immunoassay

A modified commercially available antigen competition enzyme immunoassay (EIA) from Cayman Chemical Company (Ann Arbor, MI) was used for the quantitation of SP, as previously described [21].

### Pharmacokinetics

A validated, liquid chromatography-tandem mass spectrometry method was utilized for the quantification of aprepitant in HIV-infected patients [22]. Both non-compartmental and population-based (nonlinear mixed effect modeling) analyses were conducted on aprepitant plasma concentration-time data. Non-compartmental results are presented herein. Peak (C_max_, T_max_) and exposure metrics (AUC) were calculated using WinNonlin version 5.2 (Pharsight Corporation). PK data were summarized with descriptive statistics and graphical presentation was made using GraphPad Prizm version 4.

### Viral Tropism

Viral tropism was assessed using the Trofile™ assay. Samples were shipped to Monogram Biosciences Inc., San Francisco, CA at day 0 and 14 of the study to evaluate any changes in the tropism of the HIV virus of the participants.

### Objectives

Our primary objectives of the study were to assess the safety and tolerability of two different doses of aprepitant for 2 weeks in HIV infected individuals and to assess the response of plasma HIV-1 RNA. Our secondary objectives were to evaluate the dose-response and pharmacokinetic and pharmacodynamic relationship between viral RNA change and aprepitant plasma levels, the effects on CD4+ and CD8+ T-cell counts, circulating SP levels, , the effects of aprepitant on viral tropism and to provide preliminary description of any change mediated by aprepitant in sleep quality, anxious mood, depressed mood and neurocognitive measures (using the Hamilton-17 Depression Rating Scale (HAM-D-17), the Hamilton Anxiety Scale (HAM-A) and the Pittsburgh Sleep Quality Index Score.

### Subjects

We included HIV infected individuals, older than 18 years of age, not receiving antiretroviral therapy for at least 16 weeks, with CD4 cell count greater than 350 cells/mm^3^, HIV RNA viral load greater than 2,000 copies and an R5 tropic virus (Monogram). We excluded individuals with a history of cancer and other serious illness, pregnancy, chronic hepatitis B or C infection, individuals with significant laboratory abnormalities, or were using steroids or any other immunomodulators or chemotherapy. We also excluded individuals with allergy or hypersensitivity to aprepitant.

The study was conducted at AIDS Clinical Trials Unit and the Clinical and Translational Research Center (CTRC) of the Hospital of the University of Pennsylvania in Philadelphia, PA, USA. All patients signed a written informed consent. The study was sponsored by the National Institutes of Mental Health, approved by the IRB of the University of Pennsylvania, the US Food and Drug Administration (IND#75,558), and registered in Clinical Trials.gov #NCT00428519.

### Statistical analysis

A sample size of 9 subjects within a dose group, gave us a 95% probability to observe one or more adverse events assuming the true underlying event rate within the dose group was at least 30%. We allowed the substitution of participants that did not complete the trial. The analyses of adverse events were primarily descriptive. The number and percent of subjects with an adverse event were calculated within each body system and overall for each of the three treatment groups. The geometric mean viral load (copies/ml) at baseline was calculated for each of the three treatment groups. For viral load (log_10_), the mean and 95% confidence intervals of the intra-individual difference (day 14 value minus day 0 value) was computed within each treatment group, whereas , for SP, the percent change from baseline was computed. For CD4+ cell count, the geometric mean at each visit was calculated for each of the three treatment groups. Linear mixed-effects models were fit to examine the potential change over time in viral load and CD4+ at days 0, 3, 7, 10 and 14 among the three treatment groups. The basic model has the viral load (or CD4+) as the outcome variable; and treatment group, visit, and treatment-by-visit as explanatory variables. Kruskal-Wallis nonparametric analysis of variance (ANOVA) was used to compare viral load at day 42, CD4+ at day 42, and SP at days 14 and 42 among the three treatment groups. Fisher's exact tests were used to compare the distribution of the grade 2–4 adverse events among the three treatment groups, and between the aprepitant-treated and the placebo groups. Wilcoxon signed-rank test was performed for the paired comparison of peak aprepitant concentration from 0 for each treatment group at day 1 and day 14. Wilcoxon signed-rank test was also performed for the paired comparison of peak aprepitant concentration from day 1 to 14 within each treatment group. Wilcoxon rank-sum test was used for the comparison of the difference in peak aprepitant concentration at day 1 or 14 between two aprepitant-treated groups. The P-values<0.05 were considered statistically significant. All data analyses were conducted using SAS 9.2.

## Results

Thirty subjects were enrolled with 10 being randomized into each of the three treatment groups (125 mg, 250 mg, and placebo), and 27 completed treatment (9, 8, 10 subjects in Low (125 mg daily), High (250 mg daily), and placebo (PL), respectively). 22 participants were naïve to antiretroviral therapy, eight had prior experience. Table 1 summarizes the demographic characteristics of the participants: 63% were male; 37% white; mean (SD) age 43 (9.3) years. Half of them had HIV RNA viral loads ≥20,000 copies/ml. Three subjects did not complete treatment. One (40-year-old black female) in the 125 mg group was discontinued due to prohibited concomitant medications. Two subjects are in the 250 mg group: one (41-year-old black female) was discontinued due to development of an exclusionary condition (pneumonia) and another (48-year-old white male) withdrew at the request of the subject (see Checklist S1).

**Table 1:** Demographic characteristics of study population.

	Treatment Group		
Characteristic	125 mg	250 mg	Placebo
Number of subjects	10	10	10
Gender			
Male	7(70%)	6(60%)	6(60%)
Age (years)			
Mean (SD)	43.2 (9.5)	43.4 (8.2)	42.7 (10.9)
Ethnicity			
Hispanic	0(0%)	0(0%)	1(10%)
Race			
White	3(30%)	4(40%)	4(40%)
Black	7(70%)	5(50%)	6(60%)
Others	0( 0%)	1(10%)	0( 0%)
Viral load at screening (copies/Ml)			
<20,000	5(50%)	5(50%)	5(50%)
≥20,000	5(50%)	5(50%)	5(50%)

**Figure 1:**
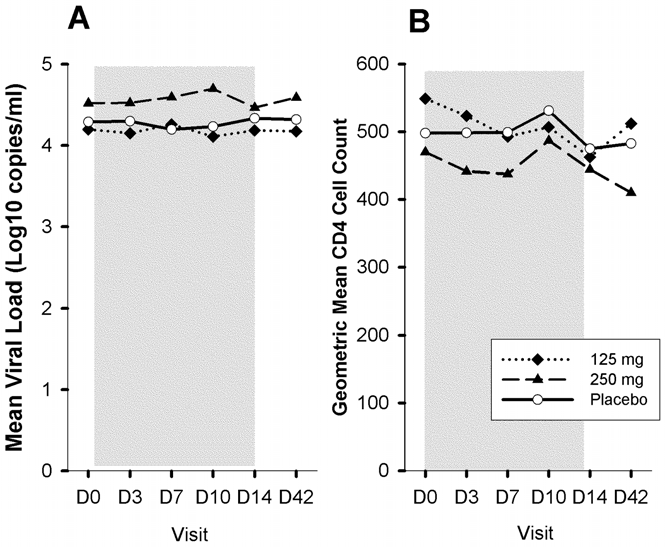
Changes in mean viral load (panel A) and CD4 cell count (panel B) by study arm. The grayed area represents the dosing period.

Geometric mean viral load (copies/ml) at baseline for 125 mg (Low), 250 mg (High), and PL was 15,709, 33,013, and 19,450 copies/mL. There were no statistically or clinically significant changes in HIV RNA viral load in any of the three groups during the drug or placebo administration (Figure 1A). The mean (95%CI) change in log10 viral load at day 14 for Low, High, and PL was −0.02(−0.24,+0.20), −0.05(−0.21,+0.10), and +0.04(−0.08,+0.16), respectively. The absolute and the CD4+ percents were fairly stable across all study visits and did not differ significantly by treatment group (Figure 1B).

Sixteen Grade 2 AEs and 1 Grade 3 AEs were reported (excluding pre-existing and ongoing events). The number of subjects with grade 2–4 adverse events (co-primary endpoint of the study) was 4(44.4%), 5(62.5%), and 1(10%) for Low, High, and PL, respectively (P = 0.056). Subjects in the aprepitant-treated groups experienced more grade 2–4 adverse events than that of the placebo group (P = 0.042). Neurological AEs were reported by 4 subjects in the High group (2 with headache, 1 with hypersomnia, and 1 with lightheadedness and dizziness) and 1 subject in the Low group (insomnia at 2 visits) (Table 2).

**Table 2:** Number of Grade 2–4* adverse events reported.

	Treatment Group		
	125 mg	250 mg	Placebo
All Grade 2–4 Events	(n = 9)	(n = 8)	(n = 10)
Number of events	5	11	1
Number (%) of subjects	4 (44.4%)	5 (62.5%)	1 (10.0%)
* Protocol-defined co-primary endpoint.			

Number (%) of Subjects with Grade 2–4 Adverse Events by Body System*								
	Treatment Group							
	125 mg(n = 9)		250 mg(n = 8)		Placebo(n = 10)		Total(n = 27)	
Body system	n	(%)	n	(%)	n	(%)	n	(%)
Gastrointestinal	1	(11.1)	0	(0.0)	0	(0.0)	1	(3.7)
Neurological	1	(11.1)	4	(50.0)	0	(0.0)	5	(18.5)
Ocular/Visual	1	(11.1)	0	(0.0)	0	(0.0)	1	(3.7)
Respiratory	0	(0.0)	0	(0.0)	1	(10.0)	1	(3.7)
Skin-Dermatological	1	(11.1)	1	(12.5)	0	(0.0)	2	(7.4)
Systemic	0	(0.0)	1	(12.5)	0	(0.0)	1	(3.7)
Uranalysis	1	(11.1)	1	(12.5)	0	(0.0)	2	(7.4)
* excludes pre-existing and ongoing events								

**Figure 2:**
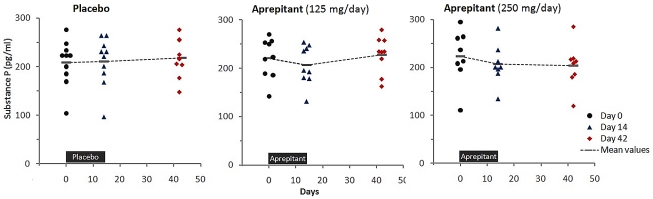
Plasma Substance P levels during the study.

Mean values of SP concentration in plasma were relatively stable in the placebo group during the study (Figure 2) but they decreased moderately in both of the groups treated with aprepitant. The mean [95% Cl] percent changes from day 0 to day 14 for the groups treated with 125 mg/day and 250 mg/day were −6.0% [−14.4, 2.4] and −5.1% [−15.9, 5.7], respectively. The decrease of the mean SP plasma levels persisted in the 250 mg arm, but was absent in the 125 mg arm at day 42. The percent changes between baseline values and the values measured at day 14 or day 42 in each of the active treatment groups were not significantly different than the changes in the placebo group. There were no statistically significant changes during the study in the Hamilton Depression (HAM-D), Anxiety (HAM-A) scores and the Pittsburgh Sleep Quality Index Score (data not shown).

**Figure 3:**
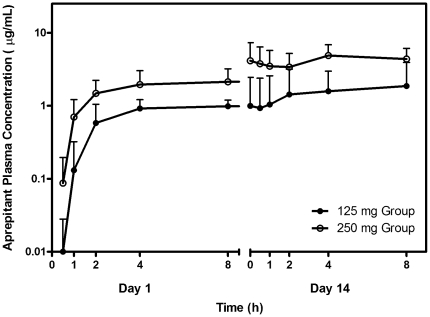
Mean aprepitant plasma concentration on days 1 and 14 of the study.

Mean (+standard deviation) aprepitant plasma concentration profiles for both dose groups are shown in Figure 3. Aprepitant plasma levels are reasonably consistent following single dose administration though somewhat more variable on day 14.

Individual peak aprepitant plasma concentrations (Cmax) on days 1 and 14 are listed in Table 3 along with the mean and standard deviation for all subjects. As expected, the aprepitant concentration significantly increased from 0 in the 125 mg dose group at day 1 (P = 0.005) and day 14 (P = 0.0077). Similarly, the aprepitant concentration significantly increased from 0 in the 250 mg dose group at day 1 (P = 0.0051) and day 14 (P = 0.012). As these data suggest, aprepitant accumulation significantly increased from day 1 to 14 in the 250 mg dose group (P = 0.012). There was not a statistically significant change from day 1 to 14 in the 125 mg dose group. Significant differences in peak aprepitant concentration were observed between the 125 and 250 mg dose groups at day 1 (P = 0.0009) and day 14 (P = 0.0039). Peak aprepitant concentrations occurred between 4 and 8 hours post dose. There were no observed shifts in Tmax with either dosing duration or amount.

**Table 3:** Maximum aprepitant plasma concentrations (µg/mL) observed on days 1 and 14 following once daily dosing of 125 and 250 mg QD to HIV-infected patients.

	Cmax: 125 mg			Cmax: 250 mg	
Sub #	Day 1	Day 14	Sub #	Day 1	Day 14
103	1.17	1.23	102	1.65	7.44
205	0.83	1.52	206	4.08	4.46
105	0.98	1.20	107	1.67	3.84
109	1.35	2.08	207	1.94	9.07
111	1.37	1.09	110	1.90	3.60
113	1.34	0.96	210	0.99	1.67
211	1.05	1.63	114	1.73	6.66
213	1.06	0.80	214	2.28	8.01
202	0.83	7.26	201	4.04	–
209	0.69	–	106	1.62	–
All Subjects			All Subjects		
Mean	1.07	1.97	Mean	2.19	5.59
SD	0.24	2.02	SD	1.04	2.57

There were no changes in the tropism of the HIV virus in study participants. All participants' viruses remained R5 tropic, as they were at the initiation of the study.

## Discussion

Aprepitant (dosed at 125 and 250 mg daily for two weeks) was safe in patients with HIV infection not receiving antiretroviral therapy. Aprepitant showed biological activity, but not clinically (or statistically) significant antiviral or immunologic improvement at this dose range and duration of therapy. Adverse events were more common in the aprepitant-treated groups, but the safety profile was similar to patterns and frequency observed that in HIV-uninfected patients treated for chemotherapy induced nausea and vomiting.

We observed moderate declines of uncertain clinical or a statistical significance in SP in both treated groups. SP enhances inflammatory cytokine (TNF-a, IL-1 and IL-6) production by immune cells such as macrophages through activation of NF-kB [23].

The observed reduction of plasma SP levels is of interest, since we have previously demonstrated increased levels of SP in men [16] and women with HIV [17]

In another context, plasma SP levels have been studied in relationship to neuropsychiatric disorders. SP levels are elevated in the cerebrospinal fluid of patients with major depression [24] and in patients post-traumatic stress disorder. SP levels are further increased [24] by traumatic physiologic stimuli, but not by neutral traumatic psychologic stimuli [24]. Recently we have shown that SP has negative immunomodulatory properties in NK cell function, that are restored by aprepitant and other NK1R antabonist [25]

A significant proportion of individuals successfully treated with antiretroviral therapy maintain elevated levels of immune activation and inflammation 2–6 years after the initiation of antiretroviral therapy [26], [27], [28]. It has become clear that chronic inflammation plays a significant role in driving morbidity and mortality in antiretroviral treated, virologically suppressed, HIV infected individuals [29], [30], [31]. Patients with HIV infection, even if successfully treated also have a high frequency of neurocognitive impairment that it is associated with this residual chronic inflammation [10], [32]. The use of pharmacological agents that target this residual inflammation may have a beneficial effect and prevent the development of these long term neurocognitive complications of HIV infection.

We did not observe changes in sleep quality, anxious mood, depressed mood and neurocognitive measures. The absence of changes in these parameters most likely reflects the lack of significant pathologic characteristics at baseline in our study population rather than a lack of effect of this compound in mood and depression. Neurokinin-1 receptor antagonists originally showed promise as novel antidepressants [33]. However, large clinical trials did not reveal evidence of efficacy in depression [34].

Aprepitant pharmacokinetic results in HIV-infected patients are generally consistent with data previously published in healthy volunteers [35] and in chemotherapy induced nausea and vomiting patients (EMEND NDA [36]), albeit dosing is of shorter duration for those indications. While these results are encouraging with respect to the portability of the dose-exposure relationship over acute dosing regimens, they must also be viewed with caution when considering the higher doses and longer duration of therapy projected necessary to treat HIV-infected individuals. Upon long-term administration, as it has been shown both in animals and humans, aprepitant induces its own metabolism resulting in decreased exposure over time. Based on previous data in animals and man, this occurs beyond 3 weeks of dosing. As aprepitant displays dose- and time dependent pharmacokinetics, clearance decreases with increased dose, likely due to saturable metabolism. Absolute bioavailability decreases as the dose increases as well. The decrease in clearance with dose is larger than the decrease in bioavailability resulting in a more than proportional increase in AUC with increased dose. A more detailed description of the PK results relative to projected dose requirements in HIV-infected patients is beyond the scope of this manuscript.

We propose several explanations of why aprepitant seems to have an antiviral effect *in vitro*, however its *in vivo* effects seem to be limited:

The dose used in this study is probably too low to see an antiviral effect. The IC50 of aprepitant is approximately 5 µM in vitro (2.5 µg/ml) and the concentrations reached in this study were clearly subtherapeutic. There are a number of strategies that can be employed to boost the exposure of aprepitant to yield exposures in the therapeutic range including the co-administration of inhibitors of the p450 system such as ritonavir or cobicistat, as is done with many antiretroviral drugs. The coadministation with these boosters is expected to result in increased plasma levels of the drug due to inhibition of the induction phenomenon that has been reported in the past with extended aprepitant administration [37].

The duration of the administration of aprepitant was too short. Because anti-inflammatory compounds do not target viral proteins and act through an indirect mechanism it is not surprising that they do not show antiviral activity after the short term administration that characterize antiretroviral trials. We have recently completed an in vivo study in Rhesus macaques evaluating the antiviral activity of aprepitant in the setting of SIV infection. The study was conducted in the Non Human Primate center of Tulane University. Two groups of Rhesus macaques (n = 4 in each group) were infected with SIVmac251. One group was untreated and served as control and the other group received aprepitant (125 mg q.d.). There was a difference in viral load set point of approximately 1 log that became apparent only after 90 days of treatment with aprepitant (A. Lackner, S.D. Douglas, J. Barrett, K. Lynch personal communication).

In conclusion, at the dose used in this exploratory phase IB study, aprepitant was safe and showed biological activity but did not show significant antiviral activity. Pharmacokinetic studies showed concentrations could reach as high as 5.5 µg/ml without enzymatic induction of the aprepitant metabolism although this must be revisited over longer drug administration. Further studies evaluating larger doses and/or co-administration of aprepitant with ritonavir or cobecistat are planned. Hence, pharmacokinetic strategies are possible to achieve higher exposure targets and overcome any presumed liability due to nonlinearities in drug clearance. A study of 375 mg of aprepitant daily in HIV infected individuals is currently ongoing (clinicaltrials.gov # NCT01300988). Future studies evaluating the therapeutic window of aprepitant in Neuro AIDS patients at doses and study durations targeted to show the multimodal effects demonstrated in vitro and in preclinical models are necessary before proof-of-concept can be established. The therapeutic goals for this class of compounds will need to be defined in terms of their ability to improve both virologic and neurocognitive outcomes.

## Supporting Information

Checklist S1 **CONSORT Checklist.**
(TIF)

Protocol S1 **Trial Protocol.**
(PDF)
