# Supplementary figures and images for: A Randomized, Placebo Controlled, Double Masked Phase IB Study Evaluating the Safety and Antiviral Activity of Aprepitant, a Neurokinin-1 Receptor Antagonist in HIV-1 Infected Adults
Source: PLoS One. 2011 Sep 8;6(9):e24180. doi: 10.1371/journal.pone.0024180 (PMC3169584; doi:10.1371/journal.pone.0024180)

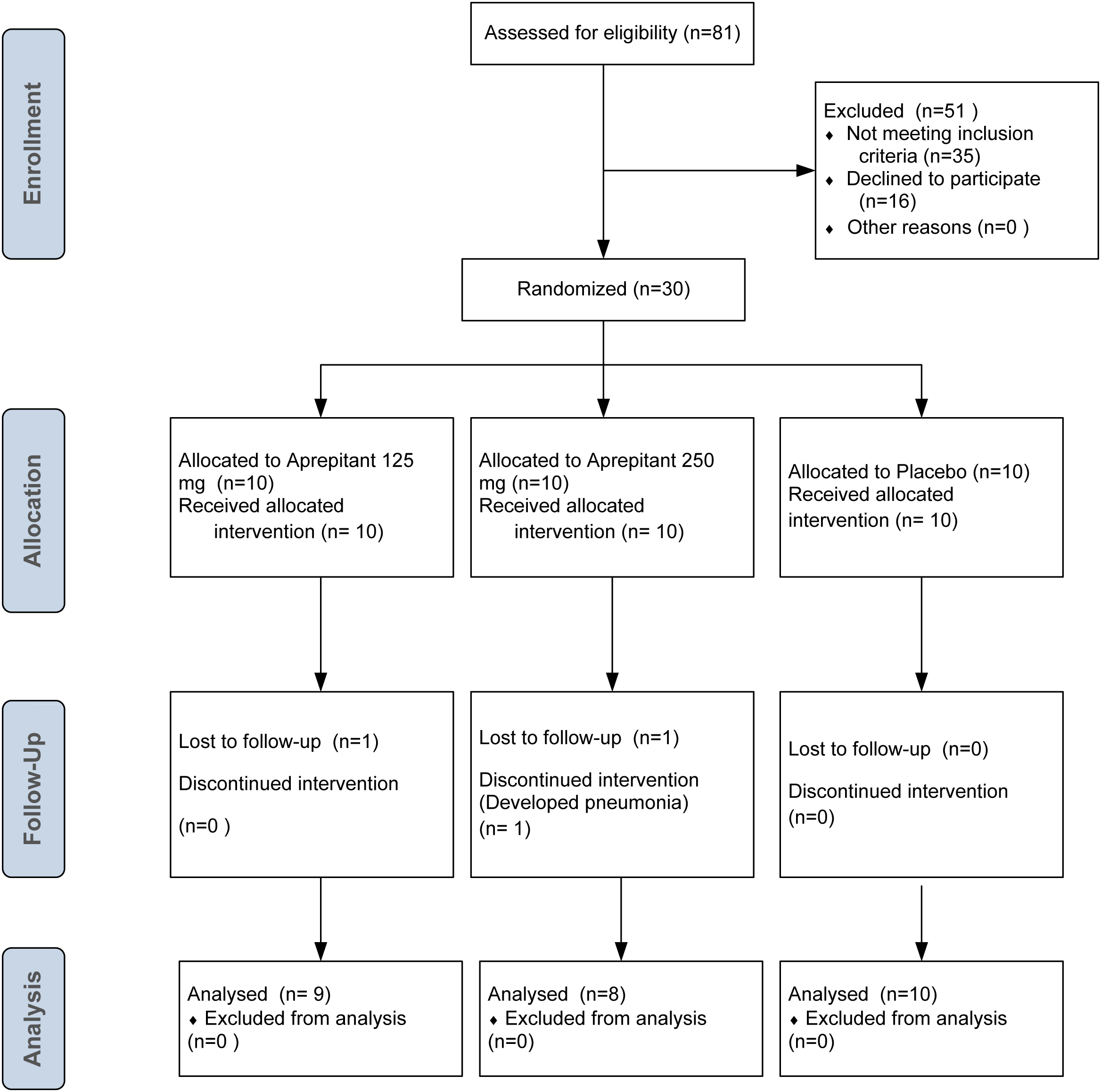

Supplement: Checklist S1 — CONSORT Checklist. (TIF) [file pone.0024180.s001.tif]
