# Supplementary material for: A Randomized, Placebo Controlled, Double Masked Phase IB Study Evaluating the Safety and Antiviral Activity of Aprepitant, a Neurokinin-1 Receptor Antagonist in HIV-1 Infected Adults
Source: PLoS One. 2011 Sep 8;6(9):e24180. doi: 10.1371/journal.pone.0024180 (PMC3169584; doi:10.1371/journal.pone.0024180)
Supplement: Protocol S1 — Trial Protocol. (PDF) [file pone.0024180.s002.pdf]

A PHASE IB, RANDOMIZED, PLACEBO CONTROLLED, DOUBLE BLIND STUDY TO DETERMINE THE SAFETY, VIRAL SUPPRESSION, PHARMACOKINETICS AND IMMUNE MODULATORY EFFECTS OF TREATMENT WITH APREPITANT (EMEND®) IN HIV INFECTED INDIVIDUALS

Sponsored by:

The National Institute of Mental Health

IND # 75,558

Version 2.0
24 January 2008

CONTENTS

Page

SITES PARTICIPATING IN THE MAIN STUDY 4
PROTOCOL TEAM ROSTER 5
SCHEMA 7

1.0 HYPOTHESIS AND STUDY OBJECTIVES 8
1.1 Hypothesis 8
1.2 Primary Objectives 8
1.3 Secondary Objectives 8

2.0 INTRODUCTION 8
2.1 Background 8
Current paradigm of HIV infection treatment 8
Limitation of current antiretroviral regimens and the need for new antiretroviral drugs 8
Substance P 9
Depression, immunity and HIV infection 10
NK cell function and HIV infection 10
2.2 Preliminary studies 11
Substance P and HIV infection 11
The importance co-receptor usage and disease progression. 12
The antiviral effects of substance P antagonists on HIV replication 13
The effects of SP and NK-1 receptor antagonists on NK cell function 14
2.3 Aprepitant pharmacology 14
Absorption and PK parameters 15
Metabolism 15
Excretion 15
Pharmacokinetics in special populations 15
2.4 Rationale 16

3.0 STUDY DESIGN 17

4.0 SELECTION AND ENROLLMENT OF SUBJECTS 17
4.1 Inclusion Criteria 17
4.2 Exclusion Criteria 18
4.3 Study Enrollment Procedures 19
4.5 Coenrollment Guidelines 20

5.0 STUDY TREATMENT 20
5.1 Regimens, Administration, and Duration 20
5.2 Product Formulation and Preparation 20
5.3 Product Supply, Distribution, and Pharmacy 21
5.4 Concomitant Medications 21
5.5 Adherence Assessment 22

TABLE OF CONTENTS (Cont'd)

Page

6.0 CLINICAL AND LABORATORY EVALUATIONS 23
6.1 Schedule of Events 23
6.2 Definitions for Schedule of Events – Timing of Evaluations 23
6.3 Special Instructions and Definitions of Evaluations 24

7.0 TOXICITY MANAGEMENT 29

8.0 CRITERIA FOR STUDY DISCONTINUATION 29

9.0 STATISTICAL CONSIDERATIONS 30
9.1 General Design Issues 30
9.2 Endpoints 30
9.3 Randomization and Stratification 31
9.4 Sample Size and Accrual 31
9.5 Monitoring 31
9.6 Analyses 32

10.0 DATA COLLECTION AND MONITORING AND ADVERSE EVENT REPORTING 36
10.1 Records to Be Kept 36
10.2 Role of Data Management 36
10.3 Clinical Site Monitoring and Record Availability 37
10.4 Expedited Adverse Event Reporting 37

11.0 HUMAN SUBJECTS 41
11.1 Institutional Review Board (IRB) Review and Informed Consent 41
11.2 Subject Confidentiality 41
11.3 Study Discontinuation 41

12.0 PUBLICATION OF RESEARCH FINDINGS 41

13.0 BIOHAZARD CONTAINMENT 41

14.0 REFERENCES 42

APPENDIX I:Karnofsky Performance Scale 48

APPENDIX II: Sample Informed Consent 49

APPENDIX III: Data Safety Monitoring Board 58

APPENDIX IV: Data Safety Monitoring Plan 59

APPENDIX V: Questionnaires 65

SITES PARTICIPATING IN THE MAIN STUDY

This is a limited Site study being conducted at Clinical Trials Unit of The Hospital of the University of Pennsylvania.

PROTOCOL TEAM ROSTER

Chair
Pablo Tebas, M.D.
Associate Professor of Medicine/Principal Investigator
University of Pennsylvania
Division of Infectious Diseases
502 Johnson Pavillion
36th Street and Hamilton Walk
Philadelphia, PA 19104--607
Phone: (215) 349-8092
Fax: (215) 615-4360
E-mail: pablo.tebas@uphs.upenn.edu

Vice Chair(s)
Steven D. Douglas MD
Professor and Associate Chair Pediatrics
Chief Section of Immunology and Director Clinical Immunology Laboratories
The Childrens Hospital of Philadelphia
34th & Civic Center Boulevard
Abramson Research Center, Room 1210
Phone: 215-590-3561
Fax: 215-590-3044
E-mail: Douglas@email.chop.edu

Statistician
Majnu John, Ph.D.
Research Statistician
Biostatistics and Data Management Core
Joseph Stokes, Jr. Research Institute
The Children's Hospital of Philadelphia and University of Pennsylvania School of Medicine
34th Street and Civic Center Blvd.
CHOP North, Suite 1035
Philadelphia, PA 19104-4399
Phone: (267) 426-5472
FAX: (215) 590-9183
E-mail: johnm@email.chop.edu

Data Manager
John Nevy
Biostatistics and Data Management Core
Joseph Stokes, Jr. Research Institute
The Children's Hospital of Philadelphia
34th Street and Civic Center Blvd.
CHOP North, Suite 1035
Philadelphia, PA 19104-4399
Phone : (215) 590-0165
Fax : (215) 590-9183
E-mail : nevy@email.chop.edu

Pharmacologist
Jeffrey S. Barrett, Ph.D., FCP
Clinical Pharmacology & Therapeutics
The Childrens Hospital of Philadelphia
Abramson Research Center, Room 907A
Philadelphia, PA 19104
Phone: (267) 426-5479
E-mail: barrettj@email.chop.edu

Virologist
Janet Lathey, Ph.D.
Director of Virology/Immunology
SeraCare, BBI Diagnostics
217 Perry Parkway
Gaithersburg, MD 20877 USA
Phone: 301-208-8100 /191
Fax: 301-208-8829
E-mail: jlathey@bbii.com

Study Coordinator
Wayne Wagner, RN
Study Coordinator
University of Pennsylvania
Division of Infectious Diseases
502 Johnson Pavilion
36th Street and Hamilton Walk
Philadelphia, PA 19104-6073
Phone: (215) 349-8092
Fax: (215) 349-8011
E-mail: wayne.wagner@uphs.upenn.edu

Site representative
Rosemarie Kappes, M.P.H.
Regulatory Manager
University of Pennsylvania
Division of Infectious Diseases
502 Johnson Pavilion
36th Street and Hamilton Walk
Philadelphia, PA 19104-6073
Phone: (215) 349-8092
Fax: (215) 349-8011
E-mail: rkappes@mail.med.upenn.edu

Medical Monitor
Ian Frank, MD
Associate Professor of Medicine
University of Pennsylvania
Division of Infectious Diseases
502 Johnson Pavillion
36th Street and Hamilton Walk
Philadelphia, PA 19104--607
Phone: (215) 662-7419
Fax: (215) 349-8011
E-mail: franki@mail.med.upenn.edu

Liaison to Integrated Pre-Clinical Clinical Project
Florin Tuluc, MD, PhD
Program Manager of the Integrated Preclinical-Clinical Project NK-1R Antagonists for HIV Therapy
The Children's Hospital of Philadelphia
34th & Civic Center Boulevard
Abramson Research Center, Room 1202A
Phone: (267)-426-5350
Fax: 215-590-3044
E-mail: tuluc@email.chop.edu

Laboratory Technologist
Nancy B. Tustin, MLT (ASCP)(HEW)
Laboratory Supervisor
The Children's Hospital of Philadelphia
34th & Civic Center Boulevard
Abramson Research Center 1204K
Philadelphia, PA 19104
Phone: 215-590-2043
Fax: 215-590-9125

Site Pharmacist
Deborah Kim , R.Ph.
ACTG Pharmacist
University of Pennsylvania Medical Center
Infectious Diseases Division
502 Johnson Pavilion
36th and Hamilton Walk
Philadelphia, PA 19104
EMail: kimd@uphs.upenn.edu
Phone: (215) 614-1900
Fax: (215) 349-8011

SCHEMA

A Phase IB, Randomized, Placebo Controlled, Double Blind Study to Determine the Safety, Viral Suppressive Potential, Pharmacokinetics and Immune Modulatory Effects of Treatment With Aprepitant (Emend) in HIV Infected Individuals

DESIGN	This is randomized, placebo controlled, double blind study to determine the safety and antiviral activity of aprepitant by comparing the change in HIV RNA viral load after 2 weeks of aprepitant monotherapy.
DURATION	42 Days
SAMPLE SIZE and POPULATION	27 HIV infected males and females ≥ 18 years old who have early infection with CD4 cell counts ≥ 350 cells/mm3.
REGIMEN	Subjects will be randomized 1:1:1 to receive two different doses of aprepitant (Emend®) or placebo. The final doses that will be used in the study will be decided according to the results obtained at the completion of the first year in project 3 of the IPCP.<br><br>Arm A: Aprepitant Placebo<br>Arm B: Aprepitant 125 mg QD<br>Arm C: Aprepitant 250 mg QD

1.0 HYPOTHESIS AND STUDY OBJECTIVES

1.1 Hypothesis

Aprepitant is safe, tolerable and has antiviral activity in HIV infected individuals.

1.2 Primary Objectives

1.2.1 To assess the safety and tolerability of aprepitant for 2 weeks at two different doses.

1.2.2 To assess the response of plasma HIV-1 RNA to two different doses of aprepitant compared with baseline.

1.3 Secondary Objectives

1.3.1 To investigate the course and duration of antiretroviral response to 2 different doses of aprepitant given over a 14-day period.

1.3.2 To evaluate the dose-response and pharmacokinetic and pharmacodynamic relationship between viral RNA change and aprepitant plasma levels

1.3.3 To evaluate aprepitant effects on CD4+ and CD8+ T-cell counts, circulating SP levels, natural killer cell number and function and CCR5 expression in peripheral PBMCs.

1.3.4 To evaluate the effects of aprepitant in the viral tropism and envelope sequence of the main HIV-1 population of the participants

1.3.5 To assess viral drug susceptibility in conjunction with baseline coreceptor tropism phenotype and changes in coreceptor phenotype after the exposure to aprepitant

1.3.6 To evaluate aprepitant effects on fasting plasma glucose, insulin, HDL, free fatty acids, and triglyceride concentrations after 14 days of treatment.

1.3.7 To provide preliminary description of any change from baseline in sleep quality, anxious mood, depressed mood and neurocognitive measures after 2 weeks of aprepitant therapy.

2.0 INTRODUCTION

2.1 Background

Current paradigm of HIV infection treatment

The current paradigm of HIV therapy is the continuous use of multiple antiretroviral drugs that target the reverse transcriptase or the protease of HIV in order to suppress plasma HIV RNA levels below the level of detection. New drugs are being developed that target the entry This strategy has been associated with a substantial decreases in the mortality associated with HIV infection, especially when used in patients with low CD4+ T cell counts [1]. This approach, however, requires a high level of adherence and is associated with a high virologic failure rate and an increasing array of long-term complications [2]. The increased awareness about these problems, together with the observational data failing to show clinical benefit when treatment is started early, treating HIV infection in patients with high CD4+ T cell counts [3-5], has had a significant impact in our approach to HIV therapy. Current antiretroviral guidelines have become more conservative, and now recommend delaying antiretroviral therapy until CD4+ T cell count levels are below 350 CD4 + T cells/mm^3 [6]. The WHO guidelines [7] for the developing world are even more conservative recommending the initiation of antiretroviral therapy for symptomatic patients or at 200 CD4 cells/mm3. This creates a unique opportunity, as many patients with high CD4 cells counts wait off antiretrovirals before initiating HAART. This antiretroviral naïve population is the population that we will use in this proof of concept study.

Limitation of current antiretroviral regimens and the need for new antiretroviral drugs

In spite of the success of antiretroviral therapy in maintaining control of HIV viral replication, treatment failure occurs in many patients. A recent study suggested that more than 50% of the individuals on treatment harbor HIV virus with evidence of resistance to one or more drugs [8].

Numerous factors contribute to treatment failure, including suboptimal adherence, side effects, suboptimal pharmacokinetics, complicated dosing regimens, drug-drug interactions, and preexisting antiretroviral drug resistance. Virologic failure leads to antiretroviral drug resistance, and cross-resistance among the currently available treatments[9].

With the number of patients experiencing failure on ART, there is a desperate need for antiretroviral agents with new mechanisms of action and activity against drug-resistant virus. Alternative paradigms for the treatment of HIV infection also need to be explored. The use of immunomodulators acting at the cellular level, rather than at the HIV virus level might be useful in delaying the need to initiate antiretroviral therapy or prolong the time off antiretrovirals in intermittent antiretroviral CD4 driven treatment strategies. The use of neurokinin-1 receptors (substance P preferring) antagonists in the management of HIV infection can serve both purposes, as these compounds may have both antiviral and immunomodulatory effects. This is the group of compounds that we will focus our efforts in this project.

Substance P

Substance P is a member of the tachykinin family[10], an undecapeptide neuropeptide secreted by neurons both in the central and the peripheral nervous system, especially in neurons that innervate the brainstem nucleus tractus solitarius and the area postrema, two areas intimately involved in the induction of vomiting. There are three known members of this group: substance P, neurokinin (NK) A, and NK B. The effects of substance P are mediated through the neurokinin-1 (NK1) receptor, a member of the G protein-coupled receptor superfamily.

Substance P also plays a central modulator role in neuroimmunoregulation, in particular, the immune functions of mononuclear phagocytes, but other immune cells including lymphocytes, neutrophils and mast cells are also able to produce this molecule and had specific receptors for it in their surfaces and a variety of effects have been elicited in vitro [11]. There are complex bidirectional interactions between the immune system and the central nervous system (recently reviewed by Steinman[12]). These two elaborate systems both sense danger and mount a counterattack to potential threats for individual. Substance P is one of the many molecules involved in this complex process.
Substance P significantly influences the host response to a variety of viral pathogens. Infection with RSV and parinfluenza virus induce the production of substance P and contribute to the pathologic effects of those organisms [13]. Mice treated with substance P antibodies with anti F glycoprotein before and during RSV infections had a shorter course of disease and prompt reduction of pulmonary inflammatory cell infiltration and decreased the number of cells expressing proinflammatory cytokines [14].

In humans substance P has been implicated in depression [15] and anxiety. Outside the CNS the gut is very rich in substance P expressing neurons where it has been suggested that they play a dual role, transmitting nocioceptive information to the brain and regulating the inflammatory and immune response to that information [16]. Substance P has also been implicated in various chronic inflammation conditions like asthma [17], pancreatitis [18] rheumatoid arthritis, inflammatory bowel disease and ulcerative colitis [19, 20].

Supporting the notion that substance P plays an important role in human physiology are the results of studies using substance P antagonists. Several substance P antagonists, peptidic and non peptidic, have been developed [21], and one of them, apprepitant has been FDA approved as an antiemetic (see below). Substance P antagonists have been studied as antidepressants [15, 22-27], and for multiple other indications [21]. Recent preclinical data, as well as

relevant clinical findings, support the potential therapeutic value of NK1 receptor antagonist also as analgesic anti-inflammatories in diseases like asthma, irritable bowel syndrome and cystitis, and even as tumor suppressants and haematopoietic agents [28]. These large clinical data sets showed an excellent safety profile, supporting the use of these compounds in HIV infected individuals.

Depression, immunity and HIV infection

The prevalence of depression and other psychiatric disorders is very high among HIV infected individuals [29]. Depression and stress have been associated in multiple studies with HIV disease progression, CD4 decline[30-32] and mortality [33, 34]. However other studies including the Multicenter AIDS Cohort Study did not observe this association, perhaps attributable to more limited psychiatric assessments [35]. Depression has also been associated with decreased adherence to HAART, the main predictor of treatment success [36, 37].

Stress and depression have has been associated with decreased natural killer (NK) cell function in HIV infected men [38, 39] and women[40]. Individuals with depression have significant reductions in natural killer cell activity, increases in activated CD8 and increases in HIV RNA viral load, markers that have been associated with an increased likelihood of disease progression[41, 42]. These immunologic effects of depression may be the important biological links that are related to the associations between altered NK function and HIV progression.

NK-1R antagonists have a significant impact in depressive symptoms and are being explored as a new class of drugs for the treatment of depression [15, 22-27]. As depressive symptoms are clearly associated with NK cell dysfunction we want to explore the impact of NK-1R antagonists in NK cell function in HIV infected individuals. This clinical study, the first that will administer NK-1R antagonists to HIV infected individuals, men and women, provides a unique opportunity to investigate these pathways of HIV immunopathogenesis.

NK cell function and HIV infection

NK cells are lymphocytes of the innate immune system that possess the capacity to recognize virally infected host cells using germline encoded activating receptors [43-45]. As a result they are generally an important part of the early immune response generated during a variety viral infections [46]. In the case of HIV, however, there is a notable decrease in NK cell function early after infection that persists and becomes more pronounced upon progression to AIDS [47]. Numerous experimental and clinical studies have documented this finding [48-53]. The HIV infection-associated negative effects upon NK cells are pervasive and involve both the quantity and functions of NK cells. These impairments of NK cells have been proposed to result in susceptibility to opportunistic infection and tumorigenesis as well as decreased control of HIV replication [47, 54]. In general HAART has been found to reverse the NK deficiencies found in patients [55-57], but defects persist as long as viral replication can be measured [55]. Moreover, in some studies, in particular in adolescents, reduction in NK cell numbers have been associated with antiretroviral therapy [58].

The mechanisms underlying NK cell impairments in HIV disease have been somewhat elusive. The effect of HIV may be in part due to the direct viral infection of NK cells, but this probably represents only a minor impairment, as most NK cells do not express CD4 or CCR5 [59]. Other experimentally rigorous mechanisms have been proposed and include selective maintenance of inhibitory class-I MHC molecules in infected cells as well as blockade of NK cell activating receptor functions by HIV gene products [60-63]. In particular, HIV *nef* has the ability to selectively

inhibit the expression of HLA-A and HLA-B alleles while preserving the expression of HLA-C and HLA-E alleles, which are ligands for NK cell inhibitory receptors [62]. Thus, HIV infection can suppress NK cell activation systems while preserving and exploiting the function NK cell inhibitory systems.

More recent studies reflecting an improved understanding of NK cell activation have shed additional light upon the decline of NK cell function found in AIDS patients. They have shown active HIV disease (defined by virema) is associated with a selective downmodulation of certain NK cell activating receptors; the natural cytotoxicity receptors (NCR) NKp30, NKp44 and NKp46 [64, 65]. In contrast, the expression and function of NK cell inhibitory receptors are preserved [65]. Since NK cells are not a major target for HIV infection, the mechanism underlying the suppression of NCR expression is probably indirect and has been proposed to be due to soluble factors.

2.2 Preliminary studies

Substance P and HIV infection

Human immune cells express SP and its receptor [66]. We have shown that HIV infection up-regulates SP expression in mononuclear phagocytes and T lymphocytes isolated from human peripheral and placental cord blood [67]. Binding of HIV gp120 to CD4 receptors on macrophages is sufficient for up-regulation of SP mRNA expression suggesting that gp120 shed from HIV-infected macrophages may play an important role in HIV-induced SP expression in these cells. The presence of substance P seems to augment the ability of the HIV virus to replicate, both in vitro [68, 69]and in vivo[58].

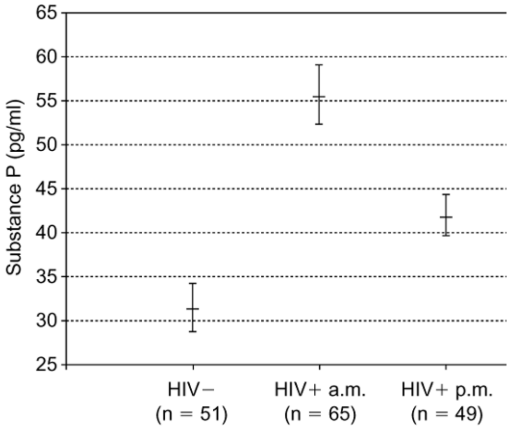

Figure 1. Substance P levels are twice as high in HIV infected subjects than HIV (-) controls [58].

Several studies have demonstrated that SP and its receptor, NK-1R, may be important and have modulatory effects in HIV infected individuals. Azzari et al. demonstrated in a small study that HIV positive children have higher levels of substance P than HIV-negative children, and that the levels of substance P correlate with immunoglobulin levels [70]. Our group has demonstrated elevated plasma levels of SP in HIV-positive men in comparison with high-risk HIV-negative men [58] (see Figure 1), and more recently in HIV infected women (unpublished results, S.D. Douglas)

SP enhanced HIV replication in human blood-isolated mononuclear phagocytes, whereas the

nonpeptide SP antagonist (CP-96,345) potently inhibited HIV infectivity of these cells in a concentration-dependent fashion [69] (See below). The mechanism/s that mediates the antiviral activity of SP antagonists are only partially understood. They may involve chemokine receptor expression[69]. Substance P antagonist decrease the expression of CCR5 in macrophage cells, a mechanism that may explain, at least in part the anti HIV activity of these compounds[69]. Other groups have recently shown that the addition of substance P increases the expression of CCR5 in an in vitro system of Dendritic/langerhans cells[71].

The importance co-receptor usage and disease progression.

The life cycle of HIV starts with the viral entry into target cells. The first step is binding of the viral surface glycoprotein gp120 to receptors on the surface of target cells. CD4 is the primary cellular receptor for HIV. However, CD4 alone is not sufficient to mediate viral infection; other factors present in human cells are necessary for entry and requires the presence of corecetptors. CCR5 is the major co-receptor that allows cellular entry together with CD4 in macrophage tropic variants of HIV-1 [72]. A homozygous deletion of the coding sequence of the CCR5 gene from position 794 to 825 (CCR5Δ32) results in a non functional chemokine receptor that has been linked to resistance to HIV-1 infections in adults [73]. Heterozygosity for this deletion does not protect against HIV infection, but increases the survival of HIV infected individuals by delaying the progression to clinical ADS, although other studies have not confirmed this observation [74, 75]. CCR5Δ32 heterozygous individuals have decreased density and expression of CCR5 in T cells [76, 77]. The decreased expression of CCR5 in the surface of T cells probably plays a central role in the slower progression of disease among these individuals. *CCR5Δ32* is common among Caucasians (~10% allele frequency in North America) but is absent or present at a very low frequency in native African and Asian populations [78].

Depending on their co-receptor usage HIV-1 strains are classified as R5 (or macrophage tropic) if they use the CCR5 co-receptor or X4 if they use the CXCR4 co-receptor. It is well recognized that R5 viruses are preferentially transmitted (>90%) and predominate from initial infection throughout the course of disease [79, 80]. As clinical disease progresses a significant percentage of individuals change the tropism of their HIV-1 quasispecies from R5 to X4. This change in tropism is associated with a change in the phenotype of the virus from non-syncytia inducing to syncytia inducing and probably with acceleration of clinical progression [79, 80].

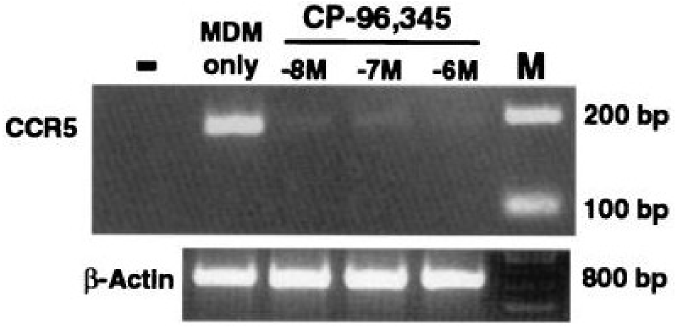

Figure 2. CP-96,345, a substance P antagonist decreased the in vitro CCR5 mRNA expression in macrophages (MDM)

Our in vitro data [69] suggest that SP antagonists will decrease the expression of CCR5 in the surface of macrophages to levels at least similar to those observed in patients heterozygous for the *CCR5Δ32* mutation. Together with a direct potential antiviral effect this could alter

disease progression. As the SP antagonist CP-96,345 seems to work at the cellular level (decreasing the expression of CCR5 mRNA and protein), rather than blocking the attachment process, it is tempting to speculate that it will be difficult to the virus to develop resistance to these compounds. Individuals heterozygous for the *CCR5Δ32* mutation do not have a significant higher frequency of X4 viruses in their system. These classes of drugs could be used as immunomodulators altering the natural history of HIV infection.

CCR5 is a promising antiviral target for HIV therapy. One of the concerns raised during the development of compounds that target this molecule is the potentially increased risk of driving the virus co receptor usage to a potentially a more pathogenic X4 strain. However, in addition to presumed natural mechanisms for prolonged selective suppression of X4 viral replication [81], CCR5 antagonists used in combination with ART should also minimize the probability of phenotypic change to X4 viruses by restricting HIV replication and reducing the accumulation of the necessary mutations.

Given this concern for chemokine receptor enhancement or change we have decided in this proof of concept study to select for individuals with relative early infection, with CD4 cell counts greater than 350 CD4 cells-mm^3, that according to the current guidelines of antiretroviral therapy would not receive treatment, and would be followed closely clinically [6]. This population of individuals represents approximately 10% of the subjects in care based in a query of our CFAR clinical database of more than 2000 individuals. Epidemiologic studies have shown that the frequency of R5 viruses at CD4 levels greater than 350 cells/mm^3 is greater than 90%. The tropism of the participants' virus will be evaluated at baseline and after 2 weeks of treatment with the SP antagonist aprepitant.. The envelope will also be sequenced to assess viral tropism and any change induced by the treatment with aprepitant.

Another potential concern of using SP antagonists and producing a significant decrease in the expression of CCR5 would be the potential immunologic effect of this. β-chemokines (MIP-1α, MIP-1β, and RANTES), are he natural ligands of these receptors, and probably serve as soluble mediators of immune responses to pathogens. Blocking CCR5 could theoretically block host responses to infection. However individuals harboring the 32-base pair deletion mutation of CCR5, which renders this receptor nonfunctional, are immunologically normal and do not have an increased risk of infection or related co morbidities; but on the other hand, the immunologic effects of decreasing the expression of CCR5 in persons who have had this receptor available previously may differ from those who lack this receptor from birth. In a four week study this phenomenon probably will not be clinically significant, but in the future, if this class of drugs is developed it will be important to monitor the occurrence of HIV-related and non-HIV-related infections in individuals treated with these compounds. In the phase II clinical trial conducted in immunosupressed oncology patients receiving chemotherapy that was used to select the final dosage of aprepitant as antiemetic there was an increase [82] in the incidence of infection in the aprepitant 125/80-mg group (13%) compared with the standard therapy group (4%). This increased risk of infection were assumed to be due to elevated dexamethasone levels as a result of the pharmacokinetic interaction [83] and have not been confirmed in the larger Phase III trials that have been conducted[84, 85]. During the phase 3 studies using aprepitant as an antidepressant for prolonged periods of time, no significant immunologic side effects were observed.

The antiviral effects of substance P antagonists on HIV replication

In a series of *in vitro* experiments we have demonstrated that SP-antagonists can inhibit HIV replication. SP-96,345, a selective NK-1 receptor binding molecule inhibits HIV Bal strain

infection of MDM in a concentration-dependent manner. We also have shown that this inhibition of HIV viral replication is mediated to the binding of CP-96,345 to the NK-1R: when the MDM were incubated with 96,344 (the inactive enantiomer of SP-96,345) the antiviral activity was lost. The combination of and anti-SP antibody and CP-96,345 had a synergistic effect on HIV replication in MDM. SP-96,345 also attenuated the TNF-α production of MDM. As already discused before SP-96,345 downregulated the CCR5 expresion in MDM. CP-96,345 inhibited the replication of HIV R5 strains (Bal, ADA, CSF-6, and BL-6) but not the X4 strain (UGO24). The neutralization of SP by anti-SP antibody resulted in down-regulation of HIV replication in MDM, and the combination of anti-SP antibody and CP-96,345 had a synergistic effect on HIV replication[69]. These findings indicate that both SP and its receptor (NK-1R) are indeed involved in HIV infection of MDM. These *in vitro* effects of the SP antagonist CP-96,345, including attenuation of TNF-α production in MDM, down-regulation of CCR5 receptor expression on MDM, and inhibition of HIV R5 strain replication are the main reasons that justify the study of this and other compounds as potential antiviral agents. Preliminary in vitro data suggest that aprepitant, the only approved substance P antagonist behaves similarly to SP-96,345 in the same in vitro system.

The effects of SP and NK-1 receptor antagonists on NK cell function

Since it has been proposed that immunosuppressive soluble factors induced during HIV disease may be responsible for depression of NK cell function we sought to determine whether SP has a role in this activity. SP is produced at increased levels during HIV infection. Patients with the highest SP levels have the most severely affected NK cell populations [50]. Further, when depression is resolved in HIV infected individuals NK cell function improves. Specifically, improvement in depressive diagnostic status and HAMD-17 score are positively associated with increases in NK cell function (p=0.0059 and p=0.042). Although these associations between HIV, depression, SP and NK cells are intriguing, they have not been investigated directly at a mechanistic level. One study has evaluated the effects of SP on NK cells, but the concentrations of SP evaluated were supraphysiologic by several logs and therefore cannot be related to our *ex vivo* observations [86].

Receptors for SP are G protein-coupled receptors of which there are two broad families known to be expressed in NK cells. These include chemokine recptors and lysophospholipid receptors. Ligands for both families of receptors are capable of inducing the motility and recruitment of, and production of cytokines by NK cells [87 88, 89]. Chemokines are also capable of enhancing NK cell cytotolytic activity. In contrast, lysophospholipids inhibit NK cell cytotoxicity [90]. Thus, although all G protein-coupled receptors have certain stimulatory activities in NK cells, there is a dichotomy as some also possess inhibitory properties. If NK-1R receptors are present in NK cells, it is possible that they may impart selective inhibitory properties under physiologic conditions. If NK-1R (SP preferring) receptors were expressed in NK cells and imparted inhibitory functions, it is likely that SP produced during HIV infection impairs NK cell function. In this case, the use of an NK-1R antagonist may serve an important immunomodulatory function for NK cells and result in increased cytotoxic functions that might improve the ability of infected individuals to fight infections and control their viral burden.

2.3 Aprepitant pharmacology

Aprepitant was approved by the Federal Drug Administration as an antiemetic in 2003. Aprepitant is a substance P preferring neurokinin 1 (NK1) receptor antagonist, the only currently approved drug of its class. Aprepitant is currently offered in two capsule formulations either 80 mg or 125 mg. This drug has been approved by the Federal Drug Administration as

an antiemetic for emesis induced by chemotherapeutic agents like cisplatin [82, 84, 85]. The main mechanism of action is via the central nervous system [91]. Aprepitant is a selective high-affinity antagonist of human substance P neurokinin 1 (NK1) receptors [92]. Aprepitant has little or no affinity for serotonin (5-HT3), dopamine, and corticosteroid receptors. Aprepitant augments the antiemetic activity of the 5-HT3-receptor antagonist ondansetron and the corticosteroid dexamethasone and inhibits both the acute and delayed phases of cisplatin induced emesis [91].

Absorption and PK parameters

Aprepitant is orally bioavailable (60 to 65%). Peak concentrations are reached after 5 hours of administration. The absorption is independent of the co-administration of food. The pharmacokinetics of aprepitant are non-linear across the clinical dose range. In healthy young adults, the increase in AUC0-¥, was 26% greater than dose proportional between 80-mg and 125-mg single doses administered in the fed state. Following oral administration of a single 125-mg dose of aprepitant on day 1 and 80 mg once daily on days 2 and 3, the AUC0-24hr was approximately 19.6 mcg.hr/mL and 21.2 mcg.hr/mL on Day 1 and Day 3, respectively. Distribution Aprepitant is greater than 95% bound to plasma proteins. The mean apparent volume of distribution at steady state (Vd,,) is approximately 70 L in humans. Aprepitant crosses the placenta in rats and rabbits and crosses the blood brain barrier in humans.

Metabolism

Aprepitant is metabolized primarily by CYP3A4 with minor metabolism by CYPIA2 and CYP2C19. Metabolism is largely via oxidation at the morpholine ring and its side chains. Seven metabolites of aprepitant, which are only weakly active, have been identified in human plasma [93-96]. This metabolic pathway suggest that aprepitant will interact favorably with ritonavir, as do many of the current available antiretrovirals.

Excretion

Following administration of a single IV 100-mg dose of [14C]-aprepitant prodrug to healthy subjects, 57% of the radioactivity was recovered in urine and 45% in feces. A study has not been conducted with radiolabeled capsule formulation. The results after oral administration may differ. Aprepitant is eliminated primarily by metabolism; aprepitant is not renally excreted.

Pharmacokinetics in special populations

The table below summarizes the known effects of aprepitant in different populations.

Table 1. Pharmacokinetics of aprepitant in different populations.

Patient population	Effects of aprepitant
Women	No difference in AUC0-24hr was observed between males and females.
Children	Not studied
Elderly	No clinically significant differences with younger patients. No dose adjustment needed

Race	Hispanics have slightly greater AUC0-24hr (approximately 25%) as compared with whites and blacks.
Moderate hepatic insufficiency	No dose adjustment needed
Renal failure	Due to modest decreases in protein binding of aprepitant in patients with renal disease, the AUC of pharmacologically active unbound drug is not significantly affected. No dosage adjustment for aprepitant is necessary for patients with renal insufficiency or for patients with ESRD undergoing hemodialysis.

2.4 Rationale

Our preliminary studies indicate that NK-1R substance P antagonists have a significant antiviral activity in vitro. This proof of concept proposal is centered on addressing the safety and antiviral activity of using aprepitant, a NK-1R substance P antagonist as an antiviral agent for the treatment of HIV infection.

The Novel HIV Therapies: Integrated Preclinical/Clinical Program (IPCP) supports (1) the discovery and preclinical development of new anti-HIV drugs and therapeutic concepts; and (2) the translation of innovative preclinical findings to the clinic via small clinical studies. This grant program funds consortia of investigators from academia and the private sector, working collaboratively on the development of a defined therapeutic concept identified and proposed by the collaborative group. This mechanism is particularly appropriate for highly experimental therapeutic strategies that are new or otherwise not yet ready for large clinical trials. The PO1-MH76388-01- project "Neurokinin1-R antagonists for HIV therapy" is a NIMH sponsored IPCP project that will evaluate the role of Neurokinin-1 receptor antagonist as potential antiretroviral agents.

Understanding the pharmacokinetics and pharmacodynamics of aprepitant in HIV infected individuals and its relationship with its potential antiviral effect is a critical part of this project.

Our preliminary studies in vitro suggest that the antiviral activity of SP antagonists might involve chemokine receptor expression[69]. Substance P antagonist decrease the expression of CCR5 in macrophage cells, a mechanism that might explain, at least in part the anti HIV activity of these compounds[69]. Our preliminary also suggest that SP may have direct effects upon NK cells as determined using a clonal NK cell line (see preliminary results section). Since we have found that SP antagonism can prevent some of this activity, it is alluring to speculate that SP antagonism may serve to reverse the impairment of NK cell function found in HIV infection. This study will provide a unique opportunity to evaluate the in vivo effects of aprepitant on CCR5 expression and message in human monocytes and lymphocytes and to study NK cell function after the administration of this drug. We will also conduct in the laboratory of Dr. Jordan Orange in vitro experiments to better understand the effects of SP and its antagonism in NK cells as well as the mechanism of these activities.

HIV infection has a significant impact of depression and anxiety disorder symptoms. In women

with HIV infection the proportion of major depression is four times higher than in HIV-negative women (19.4 vs 4.8%) [97]. This study provides a unique opportunity to explore the effects of NK-1 receptors antagonists on depression, anxiety and sleep in HIV infected subjects.

3.0 STUDY DESIGN

This is randomized, placebo controlled, double blind study to determine the antiviral activity of aprepitant by comparing the change in HIV RNA viral load after 2 weeks of aprepitant monotherapy.

27 patients with HIV-1 infection, not receiving antiretroviral therapy and with CD4+ cell count ≥ 350/mm^3 and plasma HIV-1 RNA of ≥2000 copies/mL will be stratified by viral load to 2 strata: <20,000 copies/mL vs ≥ 20,000 copies/mL and randomized within stratum 1:1:1 to receive two different doses of aprepitant (Emend®) or placebo.

Arm A: Placebo
Arm B: Aprepitant 125 mg QD
Arm C: Aprepitant 250 mg QD

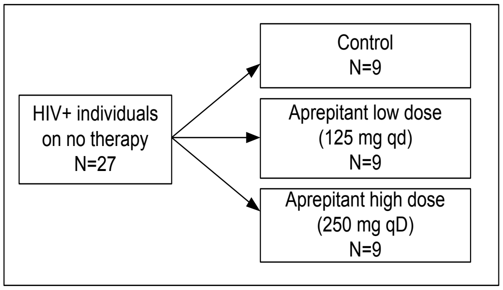

Additionally, blood samples will be collected from each study participant during the clinical phase of this study for pharmacokinetic assessment. Samples will be drawn at 0, 30 minutes, 1, 2, 4 and 8 hours after the administration of aprepitant will be collected for all subjects on days 0, and 14 of the study for pharmacokinetic analysis. Trough concentration (pre-dose) will be assessed on days 3, 7 and 10 as well. Participants will be admitted to the General Clinical Research Center of the University of Pennsylvania for the more intensive PK evaluations.

4.0 SELECTION AND ENROLLMENT OF SUBJECTS

4.1 Inclusion Criteria

4.1.1 HIV-1 infection, as documented by any licensed ELISA test kit and confirmed by Western blot at any time prior to study entry. HIV-1 culture, HIV-1 antigen, plasma HIV-1 RNA, or a second antibody test by a method other than ELISA is acceptable as an alternative confirmatory test.

4.1.2 CD4+ cell count ≥ 350/mm^3 obtained within 90 days prior to study entry and performed at any CLIA-certified laboratory.

4.1.3 Plasma HIV-1 RNA of ≥2,000 copies/mL as measured by any standard assay (the Roche Amplicor, the UltraSensitive HIV-1 Monitor assay (Roche Molecular Systems),

or Version 3 bDNA assay or other) and performed within 90 days prior to study entry by any laboratory that is CLIA-certified (or its equivalent) for the assay.

4.1.4 CCR5 tropic virus exclusively as determined by the Monogram tropism assay (PhenoSense Entry™) to be performed within 90 days of study entry.

4.1.5 Laboratory values obtained within 30 days prior to study entry, as follows:

- Absolute neutrophil count (ANC) ≥ 750/mm^3
- Hemoglobin ≥ 10.0 g/dL
- Platelet count ≥ 100,000/mm^3
- Creatinine ≤ 2 x ULN (fasting)
- AST (SGOT), ALT (SGPT), and alkaline phosphatase ≤ 2 x ULN
- Total bilirubin ≤ 2.5 x ULN
- Albumin ≥ 3 g/dL

4.1.6 Female subjects of reproductive potential must have a negative spot urine pregnancy test result (with a sensitivity of at least 50 mIU/mL) performed at entry, prior to starting initial study treatment.

4.1.7 All subjects must agree not to participate in a conception process while on study drug and for 30 days after stopping the medication.

If participating in sexual activity that could lead to pregnancy, the female study subject must use at least one of the forms of contraception listed below while receiving the protocol-specified medication and for 30 days after stopping the medication.

- Condoms (male or female) with or without a spermicidal agent
- Diaphragm or cervical cap with spermicide
- IUD

Female subjects, who are not of reproductive potential defined as women who have been post-menopausal for at least 24 consecutive months, or women who have undergone surgical sterilization, (e.g. hysterectomy, bilateral oophorectomy, or salpingotomy) are eligible without requiring the use of contraception. Subject reported history is acceptable for documentation of sterilization, other contraceptive methods, menopause and a child's reproductive potential**.**

4.1.8 Karnofsky performance score ≥ 80 within 30 days prior to study entry (Appendix I).

4.1.9 Men and women ≥ 18 years of age.

4.1.10 Ability and willingness of subject or legal guardian/representative to give written informed consent.

4.1.11 Willing to return for a follow-up visit on day 42.

4.1.12 Subjects taking any precautionary concomitant medications must be on stable doses for >8 weeks prior to study entry and have no plans to change medications or doses for the duration of the study.

4.2 <u>Exclusion Criteria</u>

4.2.1 Receipt of antiretroviral treatment within the 16 weeks prior to study entry or intent to initiate antiretroviral therapy within 60 days after entry.

4.2.2 Diabetes requiring treatment with oral hypoglycemics or insulin therapy.

4.2.3 Pregnancy within 90 days prior to study entry.

4.2.4 Breast-feeding.

4.2.5 Use of drugs that are inhibitors or inducers of metabolism by the cytochrome P450 CYP3A4 or CYP2C9 (such as warfarin and phenytoin) within 7 days of study entry.

4.2.6 Use of systemic corticosteroids or hormonal agents within 90 days prior to study entry.

4.2.7 Use of any immunomodulator, HIV vaccines, or investigational therapy within 90 days prior to study entry. However, if the experimental agent has a short half life, as determined by the Principal Investigator, the required wash out period can be reduced to 30 days.

4.2.8 Any vaccination within 30 days prior to study entry.

4.2.9 Use of systemic cytotoxic chemotherapy within 90 days prior to study entry.

4.2.10 History of allergy to aprepitant or its formulations.

4.2.11 Active drug or alcohol use or dependence that, in the opinion of the investigator, would interfere with adherence to study requirements.

4.2.12 History of chronic active hepatitis B or C infection or severe hepatic dysfunction (Child-Pugh score > 9) regardless of etiology

4.2.13 Serious illness requiring systemic treatment and/or hospitalization until subject either completes therapy or is clinically stable on therapy, in the opinion of the investigator, for at least 14 days prior to study entry.

4.2.14 Weight < 40 kg or 88 lbs. within 90 days prior to study entry.

4.2.15 History of severe psychiatric comorbidities, such as depression, schizophrenia, mania, psychosis.

,

4.3 <u>Study Enrollment Procedures</u>

4.3.1 Prior to implementation of this protocol, the site must have the protocol and consent form approved by their local institutional review board (IRB). IRB approval must occur before any subjects can be enrolled in this study.

Once a candidate for study entry has been identified, details will be carefully discussed with the subject. The subject (or legal guardian) will be asked to read and sign the consent form that was approved by the site's IRB.

4.3.2 Information on Randomization

Patients will be randomized in permuted blocks of size 3. A randomization schedule within stratum will be prepared prior to the start of the study. Sealed envelopes containing the treatment assignments will be given to the pharmacist who will retain the envelopes at all times. The pharmacist will only open the next envelope in the stratum when a patient is randomized and the pharmacist is requested to dispense study medication. Randomized treatment records will be kept with the pharmacist. All other study staff (nurse, PI, etc.) will be blinded for the duration of the trial.

If a patient is randomized, but withdraws consent prior to treatment or does not receive treatment due to any reason, the patient ID. and treatment assignment will NOT be reused.

.

4.5 Coenrollment Guidelines

Patients will not be allowed to coenroll in other antiretroviral or immunomodulator studies for the duration of this study

5.0 STUDY TREATMENT

5.1 Regimens, Administration, and Duration

Subjects will be randomized to one of three study arms: placebo, 125 mg aprepitant or 250 mg aprepitant QD for a total of 14 days.

Dose modification and treatment modification will not be allowed. No dose modification according to weight is considered for this study.

5.2 Product Formulation and Preparation

This study will provide capsules of aprepitant 125 mg and matching placebo. Aprepitant capsules will be overencapsulated with an empty, red, size,0 gelatin capsule (without lactose as filler). Matching placebo capsules will be manufactured by the site pharmacy in a designated compounding area. Lactose (supplied by Spectrum) and red, size 0 gelatin capsules distributed by Apothecary Products, will be utilized for compounding placebo capsules. The aprepitant and the placebo for aprepitant must be stored under controlled room temperature 20-25°C (68-77°F) and protected from light, humidity and excessive heat.

Once the pharmacist opens the randomization envelope and knows the randomization assignment, the pharmacist will dispense the appropriate dosage (two placebo capsules; one-125 mg aprepitant capsule + one placebo capsule; or two aprepitant 125 mg capsules). The capsules will be placed in a blister pack adherence medication card **. Only 14 days' of medication are dispensed for the duration of the study. All prescription labels will have preprinted hospital's address and the Infectious Disease CTU office phone number as well as the "Caution: New Drug. Limited By Federal Law to Investigational Use" warning. The labels will also have the following:

a. patient's name
b. SID and/or PID number
c. Rx number and date of clinic visit
d. directions for proper use

e. principal investigator's name
f. quantity dispensed
g. medication dispensed will be labeled as "Aprepitant 125 mg/placebo" to maintain the blinded treatment

**The blister pack adherence card is NOT child resistant and should be labeled as such. Patients must be counseled about keeping the medication card out of the reach of children.

5.3 ***Product Supply, Distribution, and Pharmacy***

5.3.1 Study Product Acquisition

Aprepitant 125 mg (and matching placebo) will be purchased and supplied through the ACTU pharmacy of the University of Pennsylvania. Study agent and placebo were compounded since the commercial manufacturer of aprepitant would not provide study agent and matching placebo.

5.3.2 Study Product Distribution

Study products will be available through the Infectious Disease CTU pharmacy. The Infectious Disease CTU pharmacist can obtain the study products as outlined in section 5.2.

5.3.3 Study Product Accountability

The site pharmacist is required to maintain complete records of all study products compounded and subsequently dispensed. All unused study products must be returned to the pharmacist after the study is completed or terminated.

5.3.4 Randomization Accountability

When the study is completed, all unused randomization envelopes will be returned to the study statistician for quality assurance of all randomization

.

5.4 ***Concomitant Medications***

Please refer to the study medication's most recent package insert to access additional current information on prohibited and precautionary medications.

5.4.1 Prohibited Medications

- Oral hypoglycemics and insulin therapy.
- Inhibitors of metabolism by the cytochrome P450 3A4 (i.e. Diltiazem, Ketoconazole, Clarithromycin, Ritonavir, Nelfinavir, Itraconazole, Nefazodone, Troleandomycin)
- Inducers of metabolism by the cytochrome P450 3A4 (i.e.: Rifampin, Carbamazepine, Phenytoin)
- Systemic corticosteroids (i.e. dexamethasone, methylprednisolone)
- Hormonal agents and oral contraceptives
- Immunomodulators
- Any vaccine

- Investigational therapy
- Systemic cytotoxic chemotherapy (i.e. Dosetaxel, Paclitaxel, Etopiside, Irinotecan, Ifosfamide, Imatinib, Vinorelbine, Vinblastine, Vincristine)
- Any antiretroviral agent
- Any medications that are metabolized by the CYP2C9, such as warfarin and phenytoin.

5.4.3 Precautionary Medications

- Tolbutamide
- Tolbutamide
- Midazolam
- Paroxetine

5.5 Adherence Assessment

Study medication will be dispensed at the entry visit. Beginning at the day 3 visit, adherence will be determined at each study visit using a pill count of study medication. Pill counts are not required after day 14 or after a subject discontinues treatment. The number of remaining pills and the number of missed doses since the last pill count will be recorded on the CRFs.

6.0 CLINICAL AND LABORATORY EVALUATIONS
6.1 Schedule of Events

Evaluation	Screening	Entry (Day 0)	D3	D7	D10	D14	D42	Prem Disc. Evals.
Documentation of HIV	X							
Medical/Medication History	X							
Clinical Assessments	X	X	X	X	X	X	X	X
Hematology	X	X	X	X	X	X	X	X
Chemistry	X	X	X	X	X	X	X	X
Fasting Lipid Panel		X				X	X	X
Liver Function Tests	X	X	X	X	X	X	X	X
Urinalysis	X	X	X		X	X	X	X
Pregnancy Testing	X	X				X	X	X
CD4 Cell Count and Differential	X	X	X	X	X	X	X	X
CCR5 expression		X				X	X	X
CCR5/CXCR4 Viral Tropism by the Monogram tropism assay (PhenoSense Entry™)	X					X	X	X
NK Flow Cytometry		X				X	X	X
Plasma SP levels		X				X		X
HIV-1 RNA	X	X	X	X	X	X	X	X
Envelope genotyping	X					X	X	X
Drug susceptibity phenotype assay	X					X	X	X
Stored Plasma/PBMC		X				X	X	X
Pharmacokinetic sampling		X				X		
The Hamilton-17 Depression Rating Scale (HAM-D-17)		X				X	X	X
The Hamilton Anxiety Scale (HAM-A)		X				X	X	X
Pittsburgh Sleep Quality Index Score		X				X	X	X

.

6.2 Definitions for Schedule of Events – Timing of Evaluations

6.2.1 Pre-randomization Evaluations

Occur prior to the subject taking any study medications, treatments, or interventions.

Screening
Screening evaluations to determine eligibility must be completed within 30 days of study entry unless otherwise specified. A screening log will be maintained with a screening i.d. only, and for excluded or refusing consent study candidates, the log will contain the reasons(s) for ineligibility or whether refused consent or were unable to participate due to another reason. No identifiers will be listed in the screening log and the screening i.d. will be different from the study i.d. for the study participants.

Entry
Evaluations must occur at least *24 hours* after screening evaluations unless otherwise specified. Subject must begin treatment within 48 hours of randomization.

6.2.2 On-Study Evaluations

Evaluations should occur after randomization/registration. Study visits must be scheduled on the weeks indicated in the schedule of events ± (1) day for days 0-7 and ± (3) days for days 10 - 42.

6.2.4 Treatment Discontinuation Evaluations

Subjects who permanently discontinue study treatment prior to completion of the study will have all final evaluations performed according to the schedule of evaluations.

6.3 Special Instructions and Definitions of Evaluations

6.3.1 Documentation of HIV

HIV-1 infection, as documented by any licensed ELISA test kit and confirmed by Western blot at any time prior to study entry. HIV-1 culture, HIV-1 antigen, plasma HIV-1 RNA, or a second antibody test by a method other than ELISA is acceptable as an alternative confirmatory test.

6.3.2 Medical History

A medical history must be present in source documents to include history of HIV-related and non-HIV-related diagnoses, prior anti-HIV therapies (if any), immunomodulatory therapies and vaccines, current prescription medications and lab reports concerning year of diagnosis and available viral loads or CD4 cell counts. History of opportunistic infections will be obtained. Any allergies to any medications and their formulations also must be documented.

6.3.3 Medication History

A medication history including start and stop dates for all medications taken within 30 days prior to entry must be present in source documents for each of the following:

- HIV treatment history, including antiretroviral medication, immune-based therapy, or HIV-related vaccines, including blinded study medications.
- Prescription medications for the treatment or prophylaxis of opportunistic infections.
- All prescription medications.
- Nonprescription medications
- Alternative therapies and dietary supplements

6.3.4 Concomitant Medications

All concomitant medications taken since the last report will be recorded in the source documents and CRFs.

All modifications to study drug including initial doses, subject-initiated, missed doses, and permanent discontinuation of treatment will be recorded on the CRFs at each visit. Subject-initiated and protocol-mandated modifications include both inadvertent and deliberate interruptions of study drug doses.

6.3.7 Clinical Assessments

Signs and Symptoms

At baseline, record all signs/symptoms on the CRF. For post-baseline assessments, all Grade *2* or higher signs/symptoms or any signs or symptoms that led to a change in treatment, regardless of grade, must be recorded on the CRFs.

Refer to the Division of AIDS Table for Grading Severity of Adult and Pediatric Adverse Events (DAIDS AE Grading Table), Version 1.0, December 2004.

Diagnoses
Record all diagnoses identified by the ACTG criteria for clinical events and other diseases. All confirmed and probable diagnoses made since the last visit will be recorded in the source documents and CRF, including current status at the time of study visit.

Targeted Physical Exam
For Screening, a targeted physical examination will be driven by any signs or symptoms previously identified that the subject has experienced within 30 days of entry. At entry and throughout the study, a targeted physical exam will be driven by any signs or symptoms identified that the subject has experienced since screening.

Vital Signs
Temperature, pulse, and blood pressure collected at all visits.

Height and Weight
Include weight at entry, day 14 and day 42, and height at entry only.

6.3.8 Laboratory Evaluations

At entry and throughout the study, all laboratory values, regardless of grade, must be recorded on the CRFs.

Refer to the Division of AIDS Table for Grading Severity of Adult and Pediatric Adverse Events (DAIDS AE Grading Table), Version 1.0, December 2004.

Hematology
Hemoglobin, hematocrit, red blood cells (RBC), mean corpuscular volume (MCV), white blood cell count (WBC), absolute neutrophil count (ANC), platelets

Chemistries
Serum amylase, Na, K, CL, BUN, and creatinine.

Fasting Lipid Panel with Insulin and Glucose
Triglycerides, total cholesterol, HDL, LDL, insulin

NOTE: Fasting is defined as no oral intake except water and regular prescription medicines for at least 8 hours prior to the study visit.

NOTE: All fasting glucose results are to be recorded on the CRFs, regardless of grade.

Liver/Kidney Function Tests
Urea, creatinine, total and direct bilirubin, AST (SGOT), ALT (SGPT), alkaline phosphatase, amylase

Urinalysis
Urine dipstick only

Pregnancy Test
Female subjects of reproductive potential must have a negative spot urine pregnancy test result (with a sensitivity of at least 50 mIU/mL) performed at day 0, prior to initiating study medication.

6.3.11 Immunologic Studies

Eligibility for study participation will be determined by a measurement obtained within 90 days prior to entry and performed at any CLIA-certified laboratory. If no CD4+/CD8+ results obtained within 90 days prior to study entry are available, then the subject must come in for a screening visit and have a sample drawn to determine eligibility. These results must be entered on the eligibility checklist.

NOTE: If the screening CD4+ count is < 350 cells/mm^3, the potential subject will be counseled that s/he may need ARV soon.

All CD4+/CD8+ results must be recorded on the CRFs, regardless of grade.

Beginning at screening, evaluations for CD4+ and CD8+ cell counts and subset percentages should be performed at the same CLIA certified (or equivalent) laboratory for all tests on an individual subject for comparison of the baseline calculated value and those obtained throughout the course of the study. (The baseline value is defined as the average of the screening and entry values.)

Because of the diurnal variation in CD4+ and CD8+ cell counts, determinations for individual subjects should be obtained consistently in either the morning or the afternoon throughout the study, if possible.

Note: Each time a CD4+/CD8+ measurement is obtained, the local laboratory must perform a WBC and differential from a sample obtained at the same time.

<u>CCR5 Expression</u>
See Laboratory Processing Chart (Manual of Procedures (MOP)) for further information.

<u>CCR5/CXCR4 Viral Tropism Assay</u>
See Laboratory Processing Chart (MOP) for further information.

<u>NK Flow Cytometry</u>
See Laboratory Processing Chart (MOP) for further information.

6.3.12 Virologic Studies

<u>HIV-1 RNA (real time)</u>
A commercially available assay, the Roche Amplicor HIV-1 monitor (Roche Molecular Systems, California) will be used to quantitate HIV RNA in the plasma. Since genetic variability of HIV has been shown to affect the quantitation of HIV RNA by different techniques, the version 1.5 (v1.5) will be used.

Screening HIV-1 RNA must be performed within 30 days of study entry by a laboratory that possesses a CLIA certification or equivalent.

<u>Envelope Genotyping</u>
See Laboratory Processing Chart (MOP) for further information.

<u>Drug Susceptibility Phenotype Assay</u>
See Laboratory Processing Chart (MOP) for further information.

<u>Stored PBMCs</u>
Samples will be stored for future undetermined protocol laboratory tests. See Laboratory Processing Chart (MOP) for further information.

Stored Plasma
Samples will be stored for future undetermined protocol laboratory tests. See Laboratory Processing Chart (MOP) for further information.

6.3.13 Pharmacokinetic Studies

Blood collections will be made at 0, 30 minutes, 1, 2, 4 and 8 hours after the administration of aprepitant. Trough concentration (pre-dose) will be assessed on days 3, 7 and 10. See Lab Appendix for further information.

6.3.14 Neurobehavioral Evaluations

The Hamilton-17 Depression Rating Scale (HAM-D-17)
The Hamilton-17 Depression Rating Scale (HAM-D17) [98], is an instrument designed to evaluate affective disorders of depressive type (Appendix V). It is used for quantifying the results of an interview and its value might be affected by the skill of the interviewer in eliciting the necessary information (only properly trained interviewers will be used during this study). The scale contains 17 variables measured on either a five-point or a three-point rating scale, the latter being used where quantification of the variable is either difficult or impossible. Among the variables are: depressed mood, suicide, work and loss of interest, retardation, agitation, gastro-intestinal symptoms, general somatic symptoms, hypochondriasis, loss of insight, and loss of weight. This scale has been modified to delete physical symptoms potentially related to HIV disease (HAM-D11). However in this protocol participants will have CD4 cell counts greater than 350 cells/mm^3 and no physicals symptoms associated with HIV infection, hence the HAM-D17 questionnaire will be used.

Participants that meet criteria for major depression will be referred for treatment. Any subject judged to be suicidal will be referred for immediate treatment.

The Hamilton Anxiety Scale (HAM-A)
The Hamilton Anxiety Scale (HAM-A) [99] is a rating scale developed to quantify the severity of anxiety symptomatology, often used in psychotropic drug evaluation (Appendix V). It consists of 14 items, each defined by a series of symptoms. Each item is rated on a 5-point scale, ranging from 0 (not present) to 4 (severe).

Pittsburgh Sleep Quality Index Score
The Pittsburgh Sleep Quality Index (PSQI) [100] is a self-rated questionnaire which assesses sleep quality and disturbances over a 1-month time interval (Appendix V). Nineteen individual items generate seven "component" scores: subjective sleep quality, sleep latency, sleep duration, habitual sleep efficiency, sleep disturbances, use of sleeping medication, and daytime dysfunction. The sum of scores for these seven components yields one global score.

7.0 TOXICITY MANAGEMENT

7.1.1 Toxicity Grading

Only toxicities considered to be possibly, probably, or definitely related to the study drug will be considered for toxicity management, except as noted below. Dosage reductions are not permitted.

Any subject experiencing intolerable toxicity as determined by the site study physician, a Grade ≥3 adverse event (AE), or a Grade ≥ 2 rash considered to be possibly, probably, or definitely related to study drug as defined in the Division of AIDS Table for Grading Severity of Adult and Pediatric Adverse Events (DAIDS AE Grading Table), Version 1.0, December 2004 (other than elevated lymphocyte or neutrophil counts) will be permanently discontinued from treatment. The DAIDS table can be found on the web site at: http:/rcc.tech-res-intl.com.

All Grade ≥3 toxicities for which treatment discontinuation is not mandated (those not attributed to study drug by the site study investigator) will be reviewed on a case-by-case basis by the safety monitoring committee and team.

7.1.2 Dose interruption

Subjects who develop a study drug-related Grade 3 or 4 adverse event or laboratory abnormality (with the exception of hyperglycemia, hypertriglyceridemia, hypercholesterolemia, or Grade 3 AST/ALT elevations in hepatitis co-infected individuals) should interrupt study medications.

If the abnormalities resolve to within one grade (not to exceed Grade 2) of the baseline level within 7 days of study drug interruption, reintroduction of treatment should begin.

If the abnormalities have not resolved to within one grade (not to exceed Grade 2) of he baseline level within 7 days of study drug interruption, the subject should be discontinued from the study.

8.0 CRITERIA FOR STUDY DISCONTINUATION

- Drug-related toxicity (see section 7.0).
- Development of an exclusionary condition (see section 4.2)
- Requirement for prohibited concomitant medications (see section 5.4)
- Failure by the subject to attend 3 consecutive clinic visits.
- Subject repeatedly noncompliant 3 days in a row or having missed three or more doses of study medication.
- Pregnancy or breast-feeding.
- Request by the subject to withdraw.
- Request of the primary care provider if s/he thinks the study is no longer in the best interest of the subject.

- Clinical reasons believed life threatening by the physician, even if not addressed in the toxicity management of the protocol.
- Subject judged by the investigator to be at significant risk of failing to comply with the provisions of the protocol as to cause harm to self or seriously interfere with the validity of the study results.
- Subject reaches a defined study endpoint, if applicable.
- At the discretion of the FDA, NIMH, or investigator.
- At the discretion of the DSMB based on study safety findings.

9.0 STATISTICAL CONSIDERATIONS

9.1 General Design Issues

9.2 Endpoints

9.2.1 Primary Endpoints

9.2.1.1 Virologic: Change in log_{10} HIV-1 RNA from baseline to Day 14. The baseline HIV-1 RNA will be the geometric mean of the two measurements obtained during the screening and at day 0 of the study. If only one such measurement is available, then this measurement will be the baseline value.

9.2.1.2 Safety: For adverse events occurring prior to permanent discontinuation of study: Incidence of Grade 2, 3, and 4 adverse events (using the DAIDS grading scale) by body system and by type.

9.2.2 Secondary endpoints

9.2.2.1 Pharmacokinetic: Individual pharmacokinetic parameters will be calculated by both non-compartmental and compartmental analysis. The following steady-state pharmacokinetic parameters will be calculated for each subject via non-compartmental analysis:

- C_{min}: Trough plasma aprepitant concentration.
- C_{max}: Maximum plasma concentration.
- T_{max}: Time to maximum plasma concentration.
- AUC_{ss}: Area under the plasma concentration-time curve at steady-state (based on steady-state assessment of trough concentrations or via modeling).

The following pharmacokinetic parameters will be calculated for each subject based on a population model incorporating relevant covariates (i.e., intrinsic or extrinsic factors affecting aprepitant pharmacokinetics)

- CL/F: oral clearance
- Vdp: Plasma volume of distribution
- T½: Half-life
- T_{ss}: time to reach steady-state

9.2.2.3 Immunologic

- NK cell percentage of total lymphocytes; absolute NK cell count, K562 lytic units per NK cell, expression of NKp30 and NKp46 on NK cells and IFN gamma production.
- NK cell percentage of total lymphocytes; absolute NK cell count, K562 lytic units per NK cell, expression of NKp30 and NKp46 on NK cells.
- CCR5 mRNA copy number per cell and CCR5 density by flow cytometry.
- CD4+ cell count at baseline, Days 3, 7, 10, 14, and 42.
- Time to permanent discontinuation of study treatment for any reason.
- Baseline coreceptor phenotype (CCR5, CXCR4, or mixed).
- Time to change from CCR5-only virus to mixed CCR5/CXCR4 or CXCR4-only virus as detected by the phenotype/genotype assays that will be used in this study.

9.2.2.4 Neurologic

- Hamilton-17 Depression Rating Scale (HAM-D-17) score
- Hamilton- Anxiety Symptoms score
- Pittsburgh Sleep Quality Index score

9.3 Randomization and Stratification

Subjects will be assigned with equal probability to one of the three treatment regimens using permuted blocks of size 3 within each stratum. Stratification is by viral load at screening: <20,000 copies/mL versus $\geq$ 20,000 copies/mL.

9.4 Sample Size and Accrual

This is a Phase Ib study and hence not a comparative study. For assessing the primary endpoint of change in viral load, we will use confidence intervals. With 9 patients within a group, a 95% confidence interval for the mean change in log10 HIV-1 RNA from baseline to day 14, with 90% coverage will be approximately 2 standard deviations wide. For a preliminary pilot study, this will give a sense of approximately where viral load values are expected to be after 4 weeks of treatment.

For the safety endpoint, with a group of 9 subjects within a dose group, there is 95% probability that we will observe during the study at least one adverse event of a particular kind provided that the true underlying event rate within the group is <30%. Thus, within a single dose group, we will only see events that are reasonably frequent. However, if there is an event that is unique to aprepitant and may occur at the same rate in both dose groups, any event with an underlying true rate of 15% or less, will occur with 95% confidence at least once in 18 subjects.

9.5 Monitoring

A Data Safety Monitoring Board (DSMB) will be constituted at the University of Pennsylvania and will review trial data on an annual basis, starting in October 2007. This Committee will be comprised of no less than three members who are not affiliated with the study, one of whom is a biostatistician. This committee will have a mechanism in place to immediately notify the PI regarding findings relevant to the safety of study participants, and will have criteria to recommend for stopping the study, should that become necessary. The DSMB will be provided reports that summarize demographics, enrollment and safety information. Specialized reports requested by the DSMB be prepared as required. The DSMB will produce an annual report for the IRB. This report will include:

1) Protocol title, HSC protocol number, and activation date of the study.
2) Number of patients enrolled to date on each cohort
3) Date of first and most recent patient enrollment
4) Summary of all adverse events regardless of grade 3 and attribution for to each cohort
5) Summary of any recent literature that may affect the ethics of the study.

Stopping rules

Stopping rules guidelines are based on exact 95% binomial intervals. If the lower bound of the exact 95% binomial interval exceed 20% at any point, either within a treatment cohort or across cohorts combined, the study will be suspended to further accrual pending DSMB review. So, for example if 4 of the first 5 or 6 subjects within a treatment cohort have a serious adverse event requiring discontinuation, the study will be suspended. However, if 4 of the first 7 subjects within a cohort are discontinued, the study will not be suspended, since a toxicity rate of <20% cannot be ruled out with 95% confidence. If the frequency of serious adverse events that warrants treatment discontinuation is greater than 33% (4 subjects) in any given arm, that arm will be closed. The events will include too the development of a new opportunistic infection, such as tuberculosis, a significant drop (greater than 50% of the CD4 + cell count and an increase in the HIV-RNA viral load greater than 1 log.
As the investigators will be blinded to the patient treatment assignment this will be done by the DSMB.

9.6 ***<u>Analyses</u>***

9.6.1 Primary Analysis

Although viral load will be measured twice weekly, the reason for these frequent measurements is monitoring of safety. For the purposes of assessing the primary analysis of efficacy of aprepitant in reducing viral load we will be assessing the difference between the $\log_{10}$viral load at baseline and at 4 weeks, and constructing a 95% confidence interval around this mean difference within each dose group. For the purposes of the primary analysis, if a subject discontinued prior to Day 14, the subject's last observation will be carried forward (LOCF) and will be used in lieu of the Day 14 value. The confidence interval will be compared to the confidence interval of the difference in the control/placebo group. It is expected that the confidence interval for the placebo control group will include 0, since no changes are expected in the viral load and the confidence intervals for either of the two doses will not include 0, if

aprepitant has an effect in reducing viral load.

Exact binomial confidence intervals will be calculated around the event rates for any individual adverse events that occur and for the overall rate of adverse events within each body system. For each patient the highest grade occurring adverse event within each body system will be assessed. Tables for adverse events by body system and severity of adverse event will be constructed. The distribution of the most severe adverse event rank per patient will be compared between the treatment groups using an exact Kruskal-Wallis test, to explore whether aprepitant or a particular dose of aprepitant is associated with a more severe profile of adverse events.

9.6.2 Secondary Analysis

9.6.2.1. Time trends

Since viral loads will be measured twice weekly initially and then weekly, a secondary analysis of these data will be a mixed-effects linear model with fixed effects of baseline viral load, time, treatment, and treatment by time interaction, with patient random effects for the intercept and the slope. With measurements done twice weekly, both a linear trend and a quadratic time effect can be evaluated even in this modest sample size. The variance structures will be either based on compound symmetry or autoregressive, but not unstructured. We will also explore whether adding other covariates, such as demographics or baseline CD4 counts has any impact on the model. Finally, we will add the 42 day measurement to the model, in a separate analysis.

9.6.2.2 Viral dynamics

Although the sample size is small, it may be sufficient to do some viral dynamics modeling.

9.6.2.3 Pharmacokinetics

Individual patient data will be summarized using a noncompartmental analysis (NCA) approach as well as a model-based approach. The NCA approach is based on statistical moment theory and is dependent on adequate data density within an individual subject. The NCA analysis will be analyzed using WinNonlin Professional version 4.0.1 and SAS version 9 for PC Windows.

Population pharmacokinetic model parameters (fixed and random) will be estimated via nonlinear mixed-effects modeling using NONMEM v. 5 (Globomax LLC, Hanover, MD). An appropriate compartmental model structure model will be developed for aprepitant based on the PK data collected in this study and previous pharmacokinetic experience with aprepitant in non-HIV patients (Aprepitant NDA). The effects of clinical and demographic factors on PK model parameters will be investigated using a backward stepwise elimination procedure in NONMEM. If model convergence is possible, the FOCE estimation method with eta-epsilon interaction will be employed. Model based

statistical inferences will be drawn using the Likelihood Ratio Test with a nominal p-value of 0.005 (which corresponds to a change of 7.88 in minimum objective function value for 1 degree of freedom and has been adjusted for multiple comparisons) [101].

Specific covariate effects to be investigated include indices of body size and composition, tissue distribution/binding, disease stage and severity, age, race, concomitant medications and gender. All covariate effects will be combined into a full model, which will serve as the starting point for the backward elimination procedure. The likelihood approximations used in nonlinear mixed-effects estimation methods often result in inaccurate ratios, and consequently, false-positive covariate effects [102]. If drug interaction parameters are found to be significant at the nominal p-value, a randomization test will be conducted to determine the actual significance for that parameter [102, 103].

Individual predicted PK parameter estimates based on the final model will be used to explore the relationship between various PK endpoints and clinical outcomes. Using a logistic regression, the probability of positive (or negative) outcomes will be predicted based on various PK metric expressions (i.e. Cmax, AUC). In this manner, we can explore the sensitivity of toxicity outcomes to amplitude or exposure as well as the occurrence of any time-dependent toxicities.

Clinical trial simulation will be employed to judge the projected performance of aprepitant in a phase IIa proof-of-concept trial. Design aspects to be examined will include the number of total subjects, the additional agents and regimens to be studied as part of HAART therapy, the number of plasma samples collected, the nominal sampling times for plasma concentrations, dose amounts and ranges, clinical response (viral load targets) and the likelihood of developing severe toxicities. The principal methodology employed for clinical trial simulation is Monte Carlo simulation [104]. Nonlinear mixed effect modeling may be used to generate parameter estimates (mean and variance) for PK and PK-PD models. The analysis of replicates of virtual trials will require the same statistical methodology and consideration as the single occurrence of an actual trial. These methods will be defined in the simulation analysis plan once the design options have been considered. An empirical determination of error rate will be made via a likelihood ratio test (implemented in NONMEM). A variety of graphical techniques including co-plots, MAE% (mean absolute error %) versus sample size plots, histograms (i.e., Likelihood ratio chi-square values for showing the percentage of trials falling within an interval) and box plots will be used to summarize the results of simulated trials.

The Laboratory for Applied PK-PD in the Pharmacology and Statistics Core processes all data on a PC platform (Pentium IV; 2.6 GHz processor) under Dr. Barrett's direction. A LINUX Workstation and Server are available for clinical trial simulation and population-based PK-PD analyses. Software for modeling and simulation includes: WinNonlin Professional v. 4.0 (Noncompartmental and Compartmental PK), SAS PC/Windows v. 8.0 (PK-PD, stats, data reduction), WinBUGS (Bayesian Forecasting), SPLUS (bootstrapping, graphics), NONMEM v. 5 and FORTRAN Compiler (Population PK-PD), Trial Simulator v. 2.0 (Clinical Trial Simulation).

9.6.2.4 Immunology

A descriptive analysis of the following parameters by arm at each time point will be done:

- NK cell percentage of total lymphocytes; absolute NK cell count, K562 lytic units per NK cell, expression of NKp30 and NKp46 on NK cells and IFN gamma production.
- NK cell percentage of total lymphocytes; absolute NK cell count, K562 lytic units per NK cell, expression of NKp30 and NKp46 on NK cells.
- CCR5 mRNA copy number per cell and CCR5 density by flow cytometry.
- CD4+ cell count at baseline, Days 3, 7, 10, 14, and 42.
- Time to permanent discontinuation of study treatment for any reason.
- Baseline coreceptor phenotype (CCR5, CXCR4, or mixed).
- Time to change from CCR5-only virus to mixed CCR5/CXCR4 or CXCR4-only virus as detected by the phenotype/genotype assays that will be used in this study.

For the purposes of this part of the analysis the effects of aprepitant in changing immunologic parameters will be assessing the difference between the absolute and percent counts at baseline and at 14 days, and constructing a 95% confidence interval around this mean difference within each dose group. For the purposes of this analysis, if a subject discontinued prior to Day 14, the subject's last observation will be carried forward (LOCF) and will be used in lieu of the Day 14 value. The confidence interval will be compared to the confidence interval of the difference in the control/placebo group. It is expected that the confidence interval for the placebo control group will include 0, since no changes are expected in the immunolgogic parameters in the control group and the confidence intervals for either of the two doses will not include 0, if aprepitant has an effect in reducing immunologic parameters.

A similar analysis to the one proposed for HIV RNA viral load will be conducted to evaluate time trends of immunologic parameters.

9.6.2.5 Neurology

Because of the preliminary nature of these data, it will be important to use graphics to plot the individual changes from baseline to 14 days for these subjects in the HAM-D 17 Depression Rating Scale score, the HAM-A, and the PSQI. We will also plot scatterplots for visual assessment of relationships between changes in depression, anxiety, sleep, viral load and CD4 counts. The scatterplots will be by treatment group (2 aprepitant doses and placebo). We will examine these difference scores for normality, and transform, if needed and possible, to achieve normality. As noted, this is a small study and the aim is exploratory, therefore we will not perform comparisons between the treatment arms, but rather evaluate each treatment arm separately. We will use either a one-sample t-test or the Wilcoxon Signed-Rank test to examine differences before and after treatment for each of the three treatment arms separately.

All null hypotheses are two-sided and all tests will be performed at a 0.05 significance level. We do not adjust the significance level for multiple testing because these analyses are exploratory. Although we do not expect loss to follow-up in 2 weeks of treatment and patients with CD4>350/ mm^3, if we do have any loss to follow-up, we will explore the potential effect of bias due to loss to follow-up and missing data thoroughly in a sensitivity analysis.

We will also explore the relationship between these outcome parameters and viral load, CD4 and NK function. In all cases, we will plot the baseline values with scatterplots to assess relationships visually (e.g., between HAM-D and viral load) and calculate a correlation. We will use a Pearson correlation if we were able to achieve approximate normality for all parameters. Otherwise, in order to maintain the ability to look at all parameters and relationships as a whole, we will use Spearman correlations. At the second step, once baseline correlations are assessed, we will look at the correlations between the changes from baseline of these parameters, in a similar fashion. As a third step, we will model the change in depression, anxiety or sleep outcome as a function of the change in viral load (if there was one) and the baseline score of depression, anxiety or sleep outcome in a multiple linear regression model. The multiple linear regression model will either use all subjects or at least the two treatment group subjects (depending on the scatterplots and correlations results) and use an indicator variable in the model for group membership. With this small number of subjects, we do not expect to be able to include in the model more than baseline and group memberships as a covariate as well as the single predictor variable we are focusing on. With three outcomes (depression, anxiety, sleep) and three predictors (viral load, CD4, NK function), we will have nine models. If any result is significant, it will only be considered as an indicator for the need of continuing in this line of research, but not inferential from this small study

10.0 DATA COLLECTION AND MONITORING AND ADVERSE EVENT REPORTING

10.1 Records to Be Kept

Case report forms (CRFs) will be created by the Biostatistics and Pharmacology Core of the IPCP. Subjects will not not be identified by name on any CRFs. Subjects will be identified by the patient identification number (PID) only at randomization.

10.2 Role of Data Management

Instructions concerning the recording of study data on CRFs will be provided by the Biostatistics and Pharmacology Core of the IPCP.. It will be the responsibility of the principal Investigator and the University of Pennsylvania Clinical Trials Unit Data Manager to monitor on a real time basis the individual subject records, including consent forms, CRFs, supporting data, laboratory specimen records, and medical records (physicians' progress notes, nurses' notes, individuals' hospital charts), to ensure protection of study subjects, compliance with the protocol, and accuracy and completeness of records.

10.3 Clinical Site Monitoring and Record Availability

An external certified monitor will be responsible for performing routine monitoring visit to the study site (Appendix IV, Data Safety Monitoring Plan). These monitoring visits will occur after the enrollment of the ninth, eighteenth, and last subjects to the study. The monitor will visit the clinical research unit to review the individual subject records, including consent forms, CRFs, supporting data, laboratory specimen records, and medical records (physicians' progress notes, nurses' notes, individuals' hospital charts), to ensure protection of study subjects, compliance with the protocol, and accuracy and completeness of records. In addition, the clinical trial site monitors also will inspect the site's regulatory files to ensure that all appropriate regulatory documents are present and regulatory requirements are being followed. An audit of the pharmacy will also be conducted to assure that standard operating procedures are being followed. The audit will include a review of IP storage and management.

The investigator will make study documents (e.g., consent forms, drug distribution forms, CRFs) and pertinent hospital or clinic records readily available for inspection by the University of Pennsylvania IRB, Office of Human Research of the University of Pennsylvania, the site monitors, the Food and Drug Administration (FDA), the NIMH, the Office for Human Research Protections (OHRP), for confirmation of the study data.

10.4 Expedited Adverse Event Reporting

10.4.1 Definitions

Study Drugs

1. Aprepitant
2. Placebo

Adverse Event

An ***adverse event*** (AE) is any symptom, sign, illness or experience that develops or worsens in severity during the course of the study. Intercurrent illnesses or injuries should be regarded as adverse events. Abnormal results of diagnostic procedures are considered to be adverse events if the abnormality:

- results in study withdrawal
- is associated with a serious adverse event
- is associated with clinical signs or symptoms
- leads to additional treatment or to further diagnostic tests
- is considered by the investigator to be of clinical significance

Serious Adverse Event

Adverse events are classified as serious or non-serious. A ***serious adverse event*** is any AE that is:

- fatal
- life-threatening
- requires or prolongs hospital stay
- results in persistent or significant disability or incapacity
- a congenital anomaly or birth defect

- an important medical event

Important medical events are those that may not be immediately life threatening, but are clearly of major clinical significance. They may jeopardize the subject, and may require intervention to prevent one of the other serious outcomes noted above. For example, drug overdose or abuse, a seizure that did not result in in-patient hospitalization, or intensive treatment of bronchospasm in an emergency department would typically be considered serious.

All adverse events that do not meet any of the criteria for serious should be regarded as ***non-serious adverse events***.

Expedited adverse event
Is an adverse event that meets the criteria for expedited reporting.

Adverse Event Reporting Period
The study period during which adverse events must be reported is normally defined as the period from the initiation of any study procedures to the end of the study treatment and follow-up. For this study, The expedited adverse event (EAE) reporting period will be the entire study duration for an individual subject (from study enrollment until study completion or discontinuation of the subject from study participation for any reason and at least 30 days after the last dose of study drug is given).

Preexisting Condition
A preexisting condition is one that is present at the start of the study. A preexisting condition should be recorded as an adverse event if the frequency, intensity, or the character of the condition worsens during the study period.

General Physical Examination Findings
At screening, any clinically significant abnormality should be recorded as a preexisting condition. At the end of the study, any new clinically significant findings/abnormalities that meet the definition of an adverse event must also be recorded and documented as an adverse event.

Post-study Adverse Event
All unresolved adverse events should be followed by the investigator until the events are resolved, the subject is lost to follow-up, or the adverse event is otherwise explained. At the last scheduled visit, the investigator should instruct each subject to report any subsequent event(s) that the subject, or the subject's personal physician, believes might reasonably be related to participation in this study. The investigator will notify the NIMH, FDA, IRB, and DSMB of any death or adverse event occurring at any time after a subject has discontinued or terminated study participation that may reasonably be related to this study. The NIMH, FDA, IRB, and DSMB will also be notified if the investigator should become aware of the development of cancer or of a congenital anomaly in a subsequently conceived offspring of a subject that has participated in this study.

Abnormal Laboratory Values

A clinical laboratory abnormality should be documented as an adverse event if any one of the following conditions is met:

- The laboratory abnormality is not otherwise refuted by a repeat test to confirm the abnormality
- The abnormality suggests a disease and/or organ toxicity
- The abnormality is of a degree that requires active management; e.g. change of dose, discontinuation of the drug, more frequent follow-up assessments, further diagnostic investigation, etc.

Hospitalization, Prolonged Hospitalization or Surgery

Any adverse event that results in hospitalization or prolonged hospitalization will be documented and reported as a serious adverse event unless specifically instructed otherwise in this protocol. Any condition resulting in surgery should be documented as an adverse event if the condition meets the criteria for an adverse event.

Neither the condition, hospitalization, prolonged hospitalization, nor surgery are reported as an adverse event in the following circumstances:

- Hospitalization or prolonged hospitalization for diagnostic or elective surgical procedures for a preexisting condition. Surgery should ***not*** be reported as an outcome of an adverse event if the purpose of the surgery was elective or diagnostic and the outcome was uneventful.
- Hospitalization or prolonged hospitalization required to allow efficacy measurement for the study.
- Hospitalization or prolonged hospitalization for therapy of the target disease of the study, unless it is a worsening or increase in frequency of hospital admissions as judged by the clinical investigator.

Grading Severity of Events

The Division of AIDS Table for Grading the Severity of Adult and Pediatric Adverse Events (DAIDS AE Grading Table), Version 1.0, December 2004, must be used and is available on the RCC Web site: http://rcc.tech-res-intl.com/.

10.4.2 Study Sponsor Notification by Investigator

The expedited adverse event (EAE) reporting requirements and definitions for this study and the methods for expedited reporting of adverse events (AEs) to the IRB of the University of Pennsylvania are defined in the Penn Manual for Clinical Research http://www.med.upenn.edu/pennmanual.

AEs reported on an expedited basis must be documented on the "Serious Adverse Event Report" IPCP study specific form.

The Table for Grading Adult Adverse Experiences, located on the DAIDS Regulatory Compliance Center Web site will be used: http://rcc.tech-res-intl.com The EAE reporting

period will be the entire study duration for an individual subject (from study enrollment until study completion or discontinuation of the subject from study participation for any reason).

After the end of the Protocol-defined EAE Reporting Period stated above, the site must report serious, unexpected, clinical suspected adverse drug reactions if the study site staff becomes aware of the event on a passive basis, i.e., from publicly available information.

A Serious Adverse Event (EAE) form must be completed by the investigator and faxed to the FDA per the guidelines below. The investigator will keep a copy of this EAE form on file at the study site. At the time of the initial report, the following information should be provided:

- Study identifier
- Study Center
- Subject number
- A description of the event
- Date of onset
- Current status
- Whether study treatment was discontinued
- The reason why the event is classified as serious
- Investigator assessment of the association between the event and study treatment

Within the following 48 hours, the investigator must provide further information on the serious adverse event in the form of a written narrative. This should include a copy of the completed Serious Adverse Event form, and any other diagnostic information that will assist the understanding of the event. Significant new information on ongoing serious adverse events should be provided promptly to the study sponsor

IRB Notification by Investigator
Reports of all serious adverse events (including follow-up information) must be submitted to the IRB within 10 working days. Copies of each report and documentation of IRB notification and receipt will be kept in the Clinical Investigator's binder.

FDA Notification by Sponsor
The study IND holder (Pablo Tebas, MD) shall notify the FDA by telephone or by facsimile transmission of any unexpected fatal or life-threatening experience associated with the use of the drug as soon as possible but no later than 7 calendar days from the sponsor's original receipt of the information.

If a previous adverse event that was not initially deemed reportable is later found to fit the criteria for reporting, the study sponsor will submit the adverse event in a written report to the FDA as soon as possible, but no later than 15 calendar days from the time the determination is made.

NIMH Medical officer (MO)/ Program Officer notification by Investigator
Copies of safety reports submitted to the FDA by Pablo Tebas, MD should be sent to the MO within 48 hours of FDA notification.

DSMB Notification by Investigator

The DSMB will be notified at the same time as the IRB, i.e. within 10 working days of becoming aware of the serious adverse event.

11.0 HUMAN SUBJECTS

11.1 Institutional Review Board (IRB) Review and Informed Consent

This protocol and the informed consent document (Appendix II) and any subsequent modifications will be reviewed and approved by the IRB or ethics committee responsible for oversight of the study. A signed consent form will be obtained from the subject (or parent, legal guardian, or person with power of attorney for subjects who cannot consent for themselves, such as those below the legal age). The consent form will describe the purpose of the study, the procedures to be followed, and the risks and benefits of participation. A copy of the consent form will be given to the subject, parent, or legal guardian, and this fact will be documented in the subject's record.

11.2 Subject Confidentiality

All laboratory specimens, evaluation forms, reports, and other records that leave the site will be identified by coded number only to maintain subject confidentiality. All records will be kept locked. All computer entry and networking programs will be done with coded numbers only. Clinical information will not be released without written permission of the subject, except as necessary for monitoring by IRB, the FDA, the NIMH, the OHRP, or the study DSMB.

11.3 Study Discontinuation

The study may be discontinued at any time by the IRB, the NIMH, the DSMB, the FDA, or other government agencies as part of their duties to ensure that research subjects are protected.

12.0 PUBLICATION OF RESEARCH FINDINGS

Publication of data will not identify subjects by name.

13.0 BIOHAZARD CONTAINMENT

As the transmission of HIV and other blood-borne pathogens can occur through contact with contaminated needles, blood, and blood products, appropriate blood and secretion precautions will be employed by all personnel in the drawing of blood and shipping and handling of all specimens for this study, as currently recommended by the Centers for Disease Control and Prevention and the National Institutes of Health.

All dangerous goods materials, including diagnostic specimens and infectious substances, must be transported according to the instructions detailed in the International Air Transport Association (IATA) Dangerous Goods Regulations"

14.0 REFERENCES

1. Palella FJ, Jr., Delaney KM, Moorman AC, et al. Declining morbidity and mortality among patients with advanced human immunodeficiency virus infection. HIV Outpatient Study Investigators. N Engl J Med 1998;338(13):853-60.
2. Carr A, Cooper DA. Adverse effects of antiretroviral therapy. Lancet 2000;356(9239):1423-30.
3. Phillips AN, Staszewski S, Weber R, et al. HIV viral load response to antiretroviral therapy according to the baseline CD4 cell count and viral load. Jama 2001;286(20):2560-7.
4. Hogg RS, Yip B, Chan KJ, et al. Rates of disease progression by baseline CD4 cell count and viral load after initiating triple-drug therapy. Jama 2001;286(20):2568-77.
5. Egger M, May M, Chene G, et al. Prognosis of HIV-1-infected patients starting highly active antiretroviral therapy: a collaborative analysis of prospective studies. Lancet 2002;360(9327):119-29.
6. Guidelines for the Use of Antiretroviral Agents in HIV-Infected Adults and Adolescents - March 23, 2004. http://aidsinfo.nih.gov. (Accessed 9/1/2004, at http://aidsinfo.nih.gov.)
7. Scaling up antiretroviral therapy in resource-limited settings: Treatment guidelines for a public health approach. 2003 Revision. Accessed in: http://www.who.int/3by5/publications/documents/arv_guidelines/en/. 2004. (Accessed at
8. Richman DD, Morton SC, Wrin T, et al. The prevalence of antiretroviral drug resistance in the United States. Aids 2004;18(10):1393-401.
9. Hirsch MS, Brun-Vezinet F, Clotet B, et al. Antiretroviral drug resistance testing in adults infected with human immunodeficiency virus type 1: 2003 recommendations of an International AIDS Society-USA Panel. Clin Infect Dis 2003;37(1):113-28.
10. Severini C, Improta G, Falconieri-Erspamer G, Salvadori S, Erspamer V. The tachykinin peptide family. Pharmacol Rev 2002;54(2):285-322.
11. Marriott I, Bost K. Substance P: Academic Press; 2001.
12. Steinman L. Elaborate interactions between the immune and nervous systems. Nat Immunol 2004;5(6):575-81.
13. Tripp RA, Moore D, Winter J, Anderson LJ. Respiratory syncytial virus infection and G and/or SH protein expression contribute to substance P, which mediates inflammation and enhanced pulmonary disease in BALB/c mice. J Virol 2000;74(4):1614-22.
14. Haynes LM, Tonkin J, Anderson LJ, Tripp RA. Neutralizing anti-F glycoprotein and anti-substance P antibody treatment effectively reduces infection and inflammation associated with respiratory syncytial virus infection. J Virol 2002;76(14):6873-81.
15. Kramer MS, Cutler N, Feighner J, et al. Distinct mechanism for antidepressant activity by blockade of central substance P receptors. Science 1998;281(5383):1640-5.
16. Mantyh PW, Rogers SD, Honore P, et al. Inhibition of hyperalgesia by ablation of lamina I spinal neurons expressing the substance P receptor. Science 1997;278(5336):275-9.
17. Bozic CR, Lu B, Hopken UE, Gerard C, Gerard NP. Neurogenic amplification of immune complex inflammation. Science 1996;273(5282):1722-5.
18. Bhatia M, Saluja AK, Hofbauer B, et al. Role of substance P and the neurokinin 1 receptor in acute pancreatitis and pancreatitis-associated lung injury. Proc Natl Acad Sci U S A 1998;95(8):4760-5.
19. Mantyh CR, Vigna SR, Maggio JE, Mantyh PW, Bollinger RR, Pappas TN. Substance P binding sites on intestinal lymphoid aggregates and blood vessels in inflammatory bowel disease correspond to authentic NK-1 receptors. Neurosci Lett 1994;178(2):255-9.
20. Mantyh CR, Vigna SR, Bollinger RR, Mantyh PW, Maggio JE, Pappas TN. Differential expression of substance P receptors in patients with Crohn's disease and ulcerative colitis.

Gastroenterology 1995;109(3):850-60.
21. Giardina GA, Gagliardi S, Martinelli M. Antagonists at the neurokinin receptors--recent patent literature. IDrugs 2003;6(8):758-72.
22. Rupniak NM. New insights into the antidepressant actions of substance P (NK1 receptor) antagonists. Can J Physiol Pharmacol 2002;80(5):489-94.
23. Rupniak NM. Elucidating the antidepressant actions of substance P (NK1 receptor) antagonists. Curr Opin Investig Drugs 2002;3(2):257-61.
24. Rupniak NM. Animal models of depression: challenges from a drug development perspective. Behav Pharmacol 2003;14(5-6):385-90.
25. Rupniak NM, Kramer MS. Discovery of the antidepressant and anti-emetic efficacy of substance P receptor (NK1) antagonists. Trends Pharmacol Sci 1999;20(12):485-90.
26. Kramer MS, Winokur A, Kelsey J, et al. Demonstration of the efficacy and safety of a novel substance P (NK1) receptor antagonist in major depression. Neuropsychopharmacology 2004;29(2):385-92.
27. Kramer MS. Update on Substance P (NK-1 receptor) antagonists in clinical trials for depression. Neuropeptides 2000;34(5):255.
28. Duffy RA. Potential therapeutic targets for neurokinin-1 receptor antagonists. Expert Opin Emerg Drugs 2004;9(1):9-21.
29. Bing EG, Burnam MA, Longshore D, et al. Psychiatric disorders and drug use among human immunodeficiency virus-infected adults in the United States. Arch Gen Psychiatry 2001;58(8):721-8.
30. Leserman J, Petitto JM, Gu H, et al. Progression to AIDS, a clinical AIDS condition and mortality: psychosocial and physiological predictors. Psychol Med 2002;32(6):1059-73.
31. Burack JH, Barrett DC, Stall RD, Chesney MA, Ekstrand ML, Coates TJ. Depressive symptoms and CD4 lymphocyte decline among HIV-infected men. Jama 1993;270(21):2568-73.
32. Page-Shafer K, Delorenze GN, Satariano WA, Winkelstein W, Jr. Comorbidity and survival in HIV-infected men in the San Francisco Men's Health Survey. Ann Epidemiol 1996;6(5):420-30.
33. Ickovics JR, Hamburger ME, Vlahov D, et al. Mortality, CD4 cell count decline, and depressive symptoms among HIV-seropositive women: longitudinal analysis from the HIV Epidemiology Research Study. Jama 2001;285(11):1466-74.
34. Mayne TJ, Vittinghoff E, Chesney MA, Barrett DC, Coates TJ. Depressive affect and survival among gay and bisexual men infected with HIV. Arch Intern Med 1996;156(19):2233-8.
35. Lyketsos CG, Hoover DR, Guccione M, et al. Depressive symptoms as predictors of medical outcomes in HIV infection. Multicenter AIDS Cohort Study. Jama 1993;270(21):2563-7.
36. Ammassari A, Antinori A, Aloisi MS, et al. Depressive Symptoms, Neurocognitive Impairment, and Adherence to Highly Active Antiretroviral Therapy Among HIV-Infected Persons. Psychosomatics 2004;45(5):394-402.
37. Cook JA, Cohen MH, Burke J, et al. Effects of depressive symptoms and mental health quality of life on use of highly active antiretroviral therapy among HIV-seropositive women. J Acquir Immune Defic Syndr 2002;30(4):401-9.
38. Evans DL, Leserman J, Perkins DO, et al. Stress-associated reductions of cytotoxic T lymphocytes and natural killer cells in asymptomatic HIV infection. Am J Psychiatry 1995;152(4):543-50.
39. Leserman J, Petitto JM, Perkins DO, Folds JD, Golden RN, Evans DL. Severe stress, depressive symptoms, and changes in lymphocyte subsets in human immunodeficiency virus-infected men. A 2-year follow-up study. Arch Gen Psychiatry 1997;54(3):279-85.
40. Evans DL, Ten Have TR, Douglas SD, et al. Association of depression with viral load, CD8 T lymphocytes, and natural killer cells in women with HIV infection. Am J Psychiatry 2002;159(10):1752-

9.
41. Giorgi JV, Hultin LE, McKeating JA, et al. Shorter survival in advanced human immunodeficiency virus type 1 infection is more closely associated with T lymphocyte activation than with plasma virus burden or virus chemokine coreceptor usage. J Infect Dis 1999;179(4):859-70.
42. Liu Z, Cumberland WG, Hultin LE, Kaplan AH, Detels R, Giorgi JV. CD8+ T-lymphocyte activation in HIV-1 disease reflects an aspect of pathogenesis distinct from viral burden and immunodeficiency. J Acquir Immune Defic Syndr Hum Retrovirol 1998;18(4):332-40.
43. Miller JS. The biology of natural killer cells in cancer, infection, and pregnancy. Exp Hematol 2001;29(10):1157-68.
44. Moretta L, Bottino C, Pende D, Mingari MC, Biassoni R, Moretta A. Human natural killer cells: their origin, receptors and function. Eur J Immunol 2002;32(5):1205-11.
45. Raulet DH. Interplay of natural killer cells and their receptors with the adaptive immune response. Nat Immunol 2004;5(10):996-1002.
46. Biron CA, Nguyen KB, Pien GC, Cousens LP, Salazar-Mather TP. Natural killer cells in antiviral defense: function and regulation by innate cytokines. Annu Rev Immunol 1999;17:189-220.
47. Scott-Algara D, Paul P. NK cells and HIV infection: lessons from other viruses. Curr Mol Med 2002;2(8):757-68.
48. Ljunggren K, Karlson A, Fenyo EM, Jondal M. Natural and antibody-dependent cytotoxicity in different clinical stages of human immunodeficiency virus type 1 infection. Clin Exp Immunol 1989;75(2):184-9.
49. Ullum H, Gotzsche PC, Victor J, Dickmeiss E, Skinhoj P, Pedersen BK. Defective natural immunity: an early manifestation of human immunodeficiency virus infection. J Exp Med 1995;182(3):789-99.
50. Douglas SD, Durako SJ, Tustin NB, et al. Natural killer cell enumeration and function in HIV-infected and high-risk uninfected adolescents. Aids Research and Human Retroviruses 2001;17(6):543-52.
51. Azzoni L, Papasavvas E, Chehimi J, et al. Sustained impairment of IFN-gamma secretion in suppressed HIV-infected patients despite mature NK cell recovery: evidence for a defective reconstitution of innate immunity. J Immunol 2002;168(11):5764-70.
52. Fortis C, Tasca S, Capiluppi B, Tambussi G. Natural killer cell function in HIV-1 infected patients. J Biol Regul Homeost Agents 2002;16(1):30-2.
53. Ullum H, Cozzi Lepri A, Aladdin H, et al. Natural immunity and HIV disease progression. Aids 1999;13(5):557-63.
54. Sirianni MC, Vincenzi L, Topino S, et al. NK cell activity controls human herpesvirus 8 latent infection and is restored upon highly active antiretroviral therapy in AIDS patients with regressing Kaposi's sarcoma. Eur J Immunol 2002;32(10):2711-20.
55. Weber K, Meyer D, Grosse V, Stoll M, Schmidt RE, Heiken H. Reconstitution of NK cell activity in HIV-1 infected individuals receiving antiretroviral therapy. Immunobiology 2000;202(2):172-8.
56. Sondergaard SR, Aladdin H, Ullum H, Gerstoft J, Skinhoj P, Pedersen BK. Immune function and phenotype before and after highly active antiretroviral therapy. J Acquir Immune Defic Syndr 1999;21(5):376-83.
57. Sirianni MC, Soddu S, Malorni W, Arancia G, Aiuti F, Soddus S. Mechanism of defective natural killer cell activity in patients with AIDS is associated with defective distribution of tubulin. J Immunol 1988;140(8):2565-8.
58. Douglas SD, Ho WZ, Gettes DR, et al. Elevated substance P levels in HIV-infected men. Aids 2001;15(15):2043-5.
59. Valentin A, Rosati M, Patenaude DJ, et al. Persistent HIV-1 infection of natural killer cells in

patients receiving highly active antiretroviral therapy. Proc Natl Acad Sci U S A 2002;99(10):7015-20.
60. Zocchi MR, Rubartelli A, Morgavi P, Poggi A. HIV-1 Tat inhibits human natural killer cell function by blocking L-type calcium channels. J Immunol 1998;161(6):2938-43.
61. Poggi A, Carosio R, Spaggiari GM, et al. NK cell activation by dendritic cells is dependent on LFA-1-mediated induction of calcium-calmodulin kinase II: inhibition by HIV-1 Tat C-terminal domain. J Immunol 2002;168(1):95-101.
62. Cohen GB, Gandhi RT, Davis DM, et al. The selective downregulation of class I major histocompatibility complex proteins by HIV-1 protects HIV-infected cells from NK cells. Immunity 1999;10(6):661-71.
63. Ahmad R, Sindhu ST, Tran P, et al. Modulation of expression of the MHC class I-binding natural killer cell receptors, and NK activity in relation to viral load in HIV-infected/AIDS patients. J Med Virol 2001;65(3):431-40.
64. De Maria A, Fogli M, Costa P, et al. The impaired NK cell cytolytic function in viremic HIV-1 infection is associated with a reduced surface expression of natural cytotoxicity receptors (NKp46, NKp30 and NKp44). Eur J Immunol 2003;33(9):2410-8.
65. Mavilio D, Benjamin J, Daucher M, et al. Natural killer cells in HIV-1 infection: dichotomous effects of viremia on inhibitory and activating receptors and their functional correlates. Proc Natl Acad Sci U S A 2003;100(25):15011-6.
66. Ho WZ, Lai JP, Zhu XH, Uvaydova M, Douglas SD. Human monocytes and macrophages express substance P and neurokinin-1 receptor. J Immunol 1997;159(11):5654-60.
67. Ho WZ, Lai JP, Li Y, Douglas SD. HIV enhances substance P expression in human immune cells. Faseb J 2002;16(6):616-8.
68. Ho WZ, Cnaan A, Li YH, et al. Substance P modulates human immunodeficiency virus replication in human peripheral blood monocyte-derived macrophages. AIDS Res Hum Retroviruses 1996;12(3):195-8.
69. Lai JP, Ho WZ, Zhan GX, Yi Y, Collman RG, Douglas SD. Substance P antagonist (CP-96,345) inhibits HIV-1 replication in human mononuclear phagocytes. Proc Natl Acad Sci U S A 2001;98(7):3970-5.
70. Azzari C, Rossi ME, Resti M, et al. [Changed levels of substance P and somatostatin in HIV-positive children]. Pediatr Med Chir 1992;14(6):577-81.
71. Hamzeh H, Sabido O, Misery L, Delézay O, Genin C. Substance P Increases Expression And Functionality Of CCR5 Co-Receptor On Dendritic Cells/Langerhans Cells And Their Precursors. In: 2nd International AIDS Society Conference on HIV Pathogenesis and Treatment Abstract No 462; 2003; Paris, France - July 13 - 16, 2003; 2003.
72. Deng H, Liu R, Ellmeier W, et al. Identification of a major co-receptor for primary isolates of HIV-1. Nature 1996;381(6584):661-6.
73. Liu R, Paxton WA, Choe S, et al. Homozygous defect in HIV-1 coreceptor accounts for resistance of some multiply-exposed individuals to HIV-1 infection. Cell 1996;86(3):367-77.
74. Huang Y, Paxton WA, Wolinsky SM, et al. The role of a mutant CCR5 allele in HIV-1 transmission and disease progression. Nat Med 1996;2(11):1240-3.
75. Dean M, Carrington M, Winkler C, et al. Genetic restriction of HIV-1 infection and progression to AIDS by a deletion allele of the CKR5 structural gene. Hemophilia Growth and Development Study, Multicenter AIDS Cohort Study, Multicenter Hemophilia Cohort Study, San Francisco City Cohort, ALIVE Study. Science 1996;273(5283):1856-62.
76. Venkatesan S, Petrovic A, Van Ryk DI, Locati M, Weissman D, Murphy PM. Reduced cell surface expression of CCR5 in CCR5Delta 32 heterozygotes is mediated by gene dosage, rather than by receptor sequestration. J Biol Chem 2002;277(3):2287-301.

77. Wu L, Paxton WA, Kassam N, et al. CCR5 levels and expression pattern correlate with infectability by macrophage-tropic HIV-1, in vitro. J Exp Med 1997;185(9):1681-91.
78. Zimmerman PA, Buckler-White A, Alkhatib G, et al. Inherited resistance to HIV-1 conferred by an inactivating mutation in CC chemokine receptor 5: studies in populations with contrasting clinical phenotypes, defined racial background, and quantified risk. Mol Med 1997;3(1):23-36.
79. Schuitemaker H, Koot M, Kootstra NA, et al. Biological phenotype of human immunodeficiency virus type 1 clones at different stages of infection: progression of disease is associated with a shift from monocytotropic to T-cell-tropic virus population. J Virol 1992;66(3):1354-60.
80. Koot M, van 't Wout AB, Kootstra NA, de Goede RE, Tersmette M, Schuitemaker H. Relation between changes in cellular load, evolution of viral phenotype, and the clonal composition of virus populations in the course of human immunodeficiency virus type 1 infection. J Infect Dis 1996;173(2):349-54.
81. Michael NL, Moore JP. HIV-1 entry inhibitors: evading the issue. Nat Med 1999;5(7):740-2.
82. Chawla SP, Grunberg SM, Gralla RJ, et al. Establishing the dose of the oral NK1 antagonist aprepitant for the prevention of chemotherapy-induced nausea and vomiting. Cancer 2003;97(9):2290-300.
83. McCrea JB, Majumdar AK, Goldberg MR, et al. Effects of the neurokinin1 receptor antagonist aprepitant on the pharmacokinetics of dexamethasone and methylprednisolone. Clin Pharmacol Ther 2003;74(1):17-24.
84. Hesketh PJ, Grunberg SM, Gralla RJ, et al. The oral neurokinin-1 antagonist aprepitant for the prevention of chemotherapy-induced nausea and vomiting: a multinational, randomized, double-blind, placebo-controlled trial in patients receiving high-dose cisplatin--the Aprepitant Protocol 052 Study Group. J Clin Oncol 2003;21(22):4112-9.
85. Poli-Bigelli S, Rodrigues-Pereira J, Carides AD, et al. Addition of the neurokinin 1 receptor antagonist aprepitant to standard antiemetic therapy improves control of chemotherapy-induced nausea and vomiting. Results from a randomized, double-blind, placebo-controlled trial in Latin America. Cancer 2003;97(12):3090-8.
86. Feistritzer C, Clausen J, Sturn DH, et al. Natural killer cell functions mediated by the neuropeptide substance P. Regul Pept 2003;116(1-3):119-26.
87. Maghazachi AA. G protein-coupled receptors in natural killer cells. J Leukoc Biol 2003;74(1):16-24.
88. Kveberg L, Bryceson Y, Inngjerdingen M, Rolstad B, Maghazachi AA. Sphingosine 1 phosphate induces the chemotaxis of human natural killer cells. Role for heterotrimeric G proteins and phosphoinositide 3 kinases. Eur J Immunol 2002;32(7):1856-64.
89. Jin Y, Knudsen E, Wang L, Maghazachi AA. Lysophosphatidic acid induces human natural killer cell chemotaxis and intracellular calcium mobilization. Eur J Immunol 2003;33(8):2083-9.
90. Maghazachi AA, Knudsen E, Jin Y, Jenstad M, Chaudhry FA. D-galactosyl-beta1-1'-sphingosine and D-glucosyl-beta1-1'-sphingosine induce human natural killer cell apoptosis. Biochem Biophys Res Commun 2004;320(3):810-5.
91. Dando TM, Perry CM. Aprepitant: a review of its use in the prevention of chemotherapy-induced nausea and vomiting. Drugs 2004;64(7):777-94.
92. Bergstrom M, Hargreaves RJ, Burns HD, et al. Human positron emission tomography studies of brain neurokinin 1 receptor occupancy by aprepitant. Biol Psychiatry 2004;55(10):1007-12.
93. Sanchez RI, Wang RW, Newton DJ, et al. Cytochrome P450 3A4 is the major enzyme involved in the metabolism of the Substance P receptor antagonist aprepitant. Drug Metab Dispos 2004.
94. Shadle CR, Lee Y, Majumdar AK, et al. Evaluation of potential inductive effects of aprepitant

on cytochrome P450 3A4 and 2C9 activity. J Clin Pharmacol 2004;44(3):215-23.
95. Huskey SE, Dean BJ, Doss GA, et al. The metabolic disposition of aprepitant, a substance P receptor antagonist, in rats and dogs. Drug Metab Dispos 2004;32(2):246-58.
96. Majumdar AK, McCrea JB, Panebianco DL, et al. Effects of aprepitant on cytochrome P450 3A4 activity using midazolam as a probe. Clin Pharmacol Ther 2003;74(2):150-6.
97. Morrison MF, Petitto JM, Ten Have T, et al. Depressive and anxiety disorders in women with HIV infection. Am J Psychiatry 2002;159(5):789-96.
98. Hamilton M. Development of a rating scale for primary depressive illness. Br J Soc Clin Psychol 1967;6:278-96.
99. Guy W. 048 HAMA Hamilton Anxiety Scale. ECDEU Assessment Manual, U.S. Department of Health and Human Services, Public Health Service - Alcohol, Drug Abuse, and Mental Health Administration. In; 1976:194-8.
100. Buysse DJ, Reynolds CF, 3rd, Monk TH, Berman SR, Kupfer DJ. The Pittsburgh Sleep Quality Index: a new instrument for psychiatric practice and research. Psychiatry Res 1989;28(2):193-213.
101. Boeckman A, Sheiner L, Beal S. NONMEM Users Guide - Part V. In; 1994.
102. Wahlby U, Jonsson EN, Karlsson MO. Assessment of actual significance levels for covariate effects in NONMEM. J Pharmacokinet Pharmacodyn 2001;28(3):231-52.
103. Gobburu JV, Lawrence J. Application of resampling techniques to estimate exact significance levels for covariate selection during nonlinear mixed effects model building: some inferences. Pharm Res 2002;19(1):92-8.
104. Holford NH, Kimko HC, Monteleone JP, Peck CC. Simulation of clinical trials. Annu Rev Pharmacol Toxicol 2000;40:209-34.

APPENDIX I: KARNOFSKY PERFORMANCE SCALE

Able to carryon normal activity; no special care is needed	100	Normal; no complaint; no evidence of disease.
	90	Able to carryon normal activity; minor signs or symptoms of disease.
	80	Normal activity with effort; some signs or symptoms of disease.
Unable to work, able to live at home and care for most personal needs; a varying amount of assistance needed	70	Cares for self, unable to carryon normal activity or to do active work.
	60	Requires occasional assistance but is able to care for most of needs.
	50	Requires considerable assistance and frequent medical care.
Unable to care for self; requires equivalent of institutional or hospital care; disease may be progressing rapidly	40	Disabled, requires special care and assistance.
	30	Severely disabled; hospitalization is indicated although death is not imminent.
	20	Very sick; hospitalization necessary; active supportive treatment is necessary.
	10	Moribund, fatal processes progressing rapidly.
	0	Dead

APPENDIX II: SAMPLE INFORMED CONSENT

UNIVERSITY OF PENNSYLVANIA HEALTH SYSTEM

A PHASE IB, RANDOMIZED, PLACEBO CONTROLLED, DOUBLE BLIND STUDY TO DETERMINE THE SAFETY, VIRAL SUPPRESSION, PHARMACOKINETICS AND IMMUNE MODULATORY EFFECTS OF TREATMENT WITH APREPITANT (EMEND®) IN HIV INFECTED INDIVIDUALS (Version 2.0, 1/24/08)

CONSENT FORM / AUTHORIZATION (HIPAA) FOR A RESEARCH STUDY

Investigators: Pablo Tebas, MD Phone Number: (215) 615-4321
Wayne Wagner, RN 215) 349-8092

24-hour emergency Number: (215) 662-6059 (ask for Infectious Diseases Fellow on call)

INTRODUCTION
You are being asked to take part in this research study because you are infected with HIV, the virus that causes AIDS, and you are not taking anti-HIV drugs (not on any antiretroviral therapy now or in the 16 weeks prior to entry or planning to start therapy in the next 60 days) and have a CD4 count of at least 350 cells. This study is sponsored by the National Institute of Mental Health (NIMH). The doctor in charge of this study at this site is Pablo Tebas, MD. Before you decide if you want to be a part of this study, we want you to know about the study.

This is a consent form. It gives you information about this study. The study staff will talk with you about this information. You are free to ask questions about this study at any time. If you agree to take part in this study, you will be asked to sign this consent form. You will get a copy to keep.

WHY IS THIS STUDY BEING DONE?
This study is being done to evaluate the anti-HIV activity (if the level of HIV in your blood can be lowered), and the safety and tolerability of the drug aprepitant. In the test tube, aprepitant has an affect on HIV and inhibits its replication. This is the first time aprepitant is being given to HIV-infected people and therefore is being used in an investigational manner (not U.S. Food and Drug Administration (FDA)-approved for HIV treatment). The FDA has approved aprepitant for the prophylactic treatment of vomiting and nausea in patients starting chemotherapy. Aprepitant has also been studied as a possible treatment for depression. This study will use two doses of aprepitant, 250 mg and 125 mg given once a day. Several questionnaires involving depression and anxiety will also be administered to see if aprepitant has any effects on these parameters.

WHAT DO I HAVE TO DO IF I AM IN THIS STUDY?

All study visits will be conducted in the General Clinic Research Center (GCRC) on 1 Dulles in the Hospital of the University of Pennsylvania.

Screening Visit

You will be asked to come to the GCRC to see if you are eligible to participate in the trial. At this screening visit, you will be asked to sign this consent form that explains the study and what will be expected of you. The study nurse will ask you questions about your medical history and medications you have taken in the past and are currently taking. You will have your vital signs and weight taken and will be asked about how you are feeling. After that you will have about 3 tablespoons of blood drawn for hematology (blood counts), chemistries (tests to see how well your liver and kidneys are working), pregnancy test (if you are a woman), HIV viral load and CD4 cell count. If there is no documentation available, a test to confirm that you are HIV positive will also be done. In addition, you will have some blood will be used for specialized immunology and virology tests, including genotype and phenotype some of the components of the HIV virus. A urinalysis will also be done.

In addition, your phone number or other contact information will be requested, so that the clinic can contact you and remind you of follow-up visits.

Entry Visit (Day 0)

You will come to the Entry visit having fasted (nothing to eat or drink 8 hours before your visit with the exception of water). You will have a brief physical exam and will be asked about any medicine changes since your last visit as well as any symptoms you may have. You will have about 7 tablespoonfuls of blood drawn for routine safety labs (hematology, chemistries with liver functions tests, fasting lipid panel), CD4 and HIV viral load test will be performed. If you are a woman able to become pregnant, you will have a pregnancy test. You will also be asked to complete three questionnaires about depression, anxiety and your sleep patterns.

You will then be randomized (as in the flipping of a coin) 2:1 to receive aprepitant or placebo (a pill that looks like the study drug but contains an inactive substance), respectively.

Therefore, you have a 66% chance of getting active drug. The study will be blind, what means that neither you, nor we, will know exactly what you are receiving. Only the pharmacist will know exactly. You could get:

- 125 mg (1 aprepitant pill plus two placebos) or 250 mg (2 aprepitant pills) of study drug by mouth per day for 14 days
- Matching Placebo (3 placebos)

A small thin tube for drawing blood samples will be placed into a vein in your arm and left in place during your stay in the clinic. You will have a pre-dose blood sample (approximately 1 teaspoon) drawn and then will take your first dose of study medication in the clinic. A small sample of blood (one teaspoon at each time point) will be taken to test for the level of drug in your blood at 30 minutes, 1 hour, 2 hours, 4 hours and 8 hours after taking the medication. The total amount of blood drawn for the PK portion of your visit will be about 2 tablespoons.

You will be given a bottle of study drug. You will bring your study pill bottle with you to all study visits.

Study Visits
After your Entry visit, you will come to the GCRC for study visits on days 3, 7, 10 and 14. You will be called a couple of days before each visit to remind you of your appointment, to come to the clinic in a fasting state for your study visit, and to bring your study pill bottles with you. The research staff will check your study pill bottle and count any remaining pills. Most importantly, you will be reminded NOT to take your study medicine in the morning of all of your study visits, as a blood sample will be drawn in the clinic to measure the level of the study drug.

Days 3, 7,and 10
At these visits, you might have a brief physical exam performed, your vital signs will be taken and you will be asked about your health and if you have any side effects from the study drug. You will also be asked about any other medicines you may be taking. Routine safety labs (chemistries and hematology), HIV viral load, and CD4 count will be checked at all visits and will require about 2 tablespoons of blood. These visits should last less than an hour.

Day 14
In addition to the procedures and laboratory evaluations done above, additional laboratory evaluations will be done at Day 14, including specialized immunology and virology testing, as well as a fasting lipid panel, pregnancy test and PK analyses.

The PK (Pharmacokinetic tests) will be performed exactly as they were at the entry visit. A small thin tube for drawing blood samples will be placed into a vein in your arm and left in place during your stay in the clinic. You will have a pre-dose blood sample drawn (approximately 1 teaspoon) and then will take your first dose of study medication in the clinic. A small sample of blood (1 teaspoon at each time point) will be taken to test for the level of drug in your blood at 30 minutes, 1 hour, 2 hours, 4 hours and 8 hours after taking the medication.

These extra tests will require about 3 tablespoons of blood, so 5 tablespoons will be drawn at these visits.

On Day 14, you will be asked to complete the same questionnaires regarding anxiety, depression and sleep patterns that you completed at the enrollment visit.

On Day 14, you can be expected to be in the GCRC for at least 8 hours; meals will be provided to you on a regular schedule to coincide with the blood draws for the PK sampling.

If you miss too many doses of study medicine, you will stop study medicine and withdraw from the study. You will be asked to complete the evaluations listed above for the Day 14 visit.

Final Study Visit (Day 42)
Finally, you will return to clinic on day 42, about 6 weeks after entering the trial. You will be asked about your health and have about 7 tablespoons of blood drawn for routine safety labs, HIV viral load, CD4, special immunology and virology samples. You will be asked to complete the same questionnaires regarding anxiety, depression and sleep patterns that you completed at the enrollment and Day 14 visits. This visit may last an hour.

HOW MANY PEOPLE WILL TAKE PART IN THIS STUDY?
About 27 people will take part in this study; 9 people in each of the groups. The University of Pennsylvania is the only center participating in this study; all 27 participants will be enrolled here.

HOW LONG WILL I BE IN THIS STUDY?
You will take the study drug for 14 days and then come in for a follow-up visit on or about day 42, which is about 6 weeks after starting the study.

WHY WOULD THE DOCTOR TAKE ME OFF THIS STUDY EARLY?
The study doctor may need to take you off the study early without your permission if:

- the study is cancelled by the U.S. Food and Drug Administration (FDA), National Institute of Mental Health (NIMH), the Safety Monitoring Committee for this study, or the University of Pennsylvania's Institutional Review Board (IRB). (An IRB is a committee that watches over the safety and rights of research subjects.)
- you are not able to attend the study visits as required by the study.

The study doctor may also need to take you off the study drug without your permission if:

- continuing the study drug may be harmful to you
- you need a treatment that you may not take while on the study
- you are not able to take the study drug as required by the study.
- you become pregnant.

If you must stop taking the study drug before the study is over, you will be asked to come in for a final visit.

WHAT ARE THE RISKS OF THE STUDY?
Aprepitant Side Effects
(Note: Adverse reaction percentages reported as part of combination chemotherapy regimen)

>10%:
Central nervous system: Fatigue (18% to 22%)
Dermatologic: hair loss (24%; placebo 22%)
Gastrointestinal: Nausea (7% to 13%), constipation (10% to 12%)
Neuromuscular & skeletal: Weakness (3% to 18%)

Miscellaneous: Hiccups (11%)

1% to 10%:

Central nervous system: Dizziness (3% to 7%)
Endocrine & metabolic: Dehydration (6%), hot flushing (3%)
Gastrointestinal: Diarrhea (6% to 10%), problems with digestion (8%), abdominal pain (5%), inflammation of the inside of your mouth (5%), stomach discomfort (4%), gastritis (4%), mucous membrane disorder (3%), throat pain (3%)
Hematologic: Neutropenia (3% to 9%), leukopenia (9%), hemoglobin decreased (2% to 5%)
Hepatic: ALT increased (6%), AST increased (3%) [AST and ALT are tests that measure the function of your liver]
Renal: BUN [measure of the function of your kidneys] increased (5%), proteinuria (7%), serum creatinine increased (4%)

Risks of Blood Draw and -IV line:

The process of drawing blood in some cases may cause bleeding, bruising, pain, blood clots, lightheadedness, and some minor swelling around the area of the needle sticks. Occasionally an infection or bleeding may develop where the needle was placed in the vein. The iv line may need to be replaced during the PK visit due to repeated blood draws. In rare instance, fainting may occur.

In addition, the multiple blood draws and visits to the research center may be time consuming and inconvenient.

ARE THERE RISKS RELATED TO PREGNANCY?

In studies with rats, no effect on fertility or embryonic development was noted. However, there have been no studies conducted in humans and therefore, if you are pregnant you will not be able to participate in this study. If you become pregnant while on study, you will be asked to stop taking the study medication.

All subjects are requested not to participate in a conception process while on study, ie. no egg or sperm donations and for all subjects able to become pregnant, use of at least one of the forms of contraception listed below while receiving the protocol-specified medication and for 30 days after stopping the medication.

- Condoms (male or female) with or without a spermicidal agent
- Diaphragm or cervical cap with spermicide
- IUD

ARE THERE BENEFITS TO TAKING PART IN THIS STUDY?

If you take part in this study, your HIV viral load may go down while you are receiving aprepitant, but it will very likely return to where it started (the baseline level) once the drug is stopped. Aprepitant may also have no effect on the HIV in your blood. You may receive no benefit from being in this study, but the information learned from this study may help others who have HIV and lead to a new way to treat HIV infection.

WHAT OTHER CHOICES DO I HAVE BESIDES THIS STUDY?
Instead of being in this study you have the choice of:

- treatment with prescription drugs available to you
- treatment with experimental drugs, if you qualify
- no treatment

Please talk to your doctor about these and other choices available to you. Your doctor will explain the risks and benefits of these choices.

WHAT ABOUT CONFIDENTIALITY?
By signing this Consent/Authorization Form you are permitting the University of Pennsylvania Health System and the School of Medicine to use your personal health information collected about you for research purposes within our institution. You are also allowing the University of Pennsylvania Health System and the School of Medicine to disclose that personal health information to outside organizations or people involved with the processing of this study.

What personal health information is collected and used in this study, and also might be shared?
Personal health and contact (phone number, address) information collected as part of this study is recorded in your clinical trial chart. This record is separate from your medical chart. Data collected for the study is reported to the study team on a case report form, which includes the information listed below, but not your name or other identifying information. Results of laboratory tests or study procedures will be copied and sent to your primary care physician by name, at your request only.

Personal health information that is collected and will be disclosed to the agencies listed on the following page as part of this research study is:

- Demographics (Race, Gender)
- Study medication compliance and toxicities
- Signs and symptoms you experience while on the study
- Current and past medical diagnoses; allergies
- Current and past medications and therapies
- Information from a physical exam: weight, blood pressure, heart rate, temperature
- Data from laboratory tests (blood chemistry and hematology tests), CD4 count, viral load, tropism assays; immunology studies; virology studies, genotyping/phenotyping studies
- Data from pharmacokinetic studies (drug levels in your blood)

Why is your personal health information being used?
Personal contact information, such as phone number and address, will be used only by clinical trial staff to get in touch with you while you are participating in this study. Your health information and results of tests and procedures are being collected as part of the research study and for the advancement of medicine and clinical care. The Principal Investigator will use the results to monitor your safety and ability to tolerate the study medications.

Which of our personnel may use or disclose your personal health information?

The following individuals and organizations may use or disclose your personal health information for this research project:

- The Principal Investigator and other University staff associated with this study;
- The University of Pennsylvania Institutional Review Boards (the Committees charged with overseeing research on human subjects) and the University of Pennsylvania Office of Regulatory Affairs
- Authorized members of the University of Pennsylvania and the University of Pennsylvania Health System and School of Medicine work force who may need to access your information in the performance of their duties, for example, to provide treatment, to ensure the integrity of the research, accounting or billing matters, etc.

Who, outside of the University of Pennsylvania Health System and the School of Medicine, might receive your personal health information?

As part of the study the Principal Investigator, study team and others listed above, may disclose your personal health information, including the results of the research study tests and procedures to the following:

- Pharmaceutical Sponsors: Drug companies (Merck Pharmaceuticals) who supply treatment for the study will have access to safety information.
- Government Agencies: Data from this study will be made available to the Food and Drug Administration and the National Institute of Mental Health, for them to evaluate the safety and efficacy of the treatments being used in this study.

Study staff will inform you if there are any changes to this list above during your active participation in the trial. Once information is disclosed to others outside the University of Pennsylvania Health System or School of Medicine the information may no longer be covered by federal privacy protection regulations.

In all disclosures outside the University of Pennsylvania Health System or School of Medicine, you will not be identified by name, social security number, address, telephone number, or any other direct personal identifier, unless disclosure of the direct identifier is required by law. Personal health information will be disclosed by a unique code number. Only study staff can break the code and identify you to your code.

How long will the University of Pennsylvania Health System and the School of Medicine be able to use or disclose your personal health information?

Your authorization for use of your personal information for this specific study does not expire. This information may be stored in a database (research repository). However, the University of Pennsylvania Health System and the School of Medicine may not re-use or re-disclose your personal health information collected for this study for another purpose other than the research described in this consent form unless you have given written permission to the Principal Investigator to do so. However, the University of Pennsylvania Institutional Review Board may grant permission to the Principal Investigator or others to use your information for another purpose after ensuring that appropriate safeguards are in place. The Institutional Review Board is a committee whose job is to protect the safety and privacy of research subjects. Results of all tests and procedures done solely

for this research study and not as part of your regular care will not be included in your medical record unless you want them to be sent to your primary care provider. You will need to complete a medical records release of information to allow us to provide study data to your doctor.

Will you be able to access your records?
You will be able to request access to your medical record when the study is completed. During your participation in the study, you will not be able to access your medical records. This will be done to prevent the knowledge of study results affecting the reliability of the study. Your information will be available should an emergency arise that would require your treating physician to know the information to best treat you. You will have access to your medical record and study information that is part of that record when the study is over. The Investigator is not required to release to you research information that is not part of your medical record.

Can you change your mind?
You may withdraw your permission for the use and disclosure of any of your personal information for research, but you must do so in writing to the Principal Investigator at 502 Johnson Pavilion. Even if you withdraw your permission, the Principal Investigator for the research study may still use your personal information that was collected prior to your written request if that information is necessary to the study. If you withdraw your permission to use your personal health information that means you will also be withdrawn from the research study.

WHAT ARE THE COSTS TO ME?
The study drug, exams, and blood tests will be provided for free.

WILL I RECEIVE ANY PAYMENT?
You will receive $25 for study visits on Days 3, 7, 10 and 42 At the entry, Day 14 visits [PK visits] you will receive $150. Thus, if you attend all study visits [$100] and have all two PK analyses performed [$300], the maximum compensation you can receive for the study is $400.

WHAT HAPPENS IF I AM INJURED?
If you have a medical emergency during the study you may contact the Principal Investigator or Emergency contact listed on page one of this form. You may also contact your own doctor, or seek treatment outside of the University of Pennsylvania. Be sure to tell the doctor or his/her staff that you are in a research study being conducted at the University of Pennsylvania. Ask them to call the telephone numbers on the first page of this consent form for further instructions or information about your care.

In the event of any physical injury resulting from research procedures, medical treatment will be provided without cost to you, but financial compensation is not otherwise available from the University of Pennsylvania. If you have an illness or injury during this research trial that is not directly related to your participation in this study, you and/or your insurance will be responsible for the cost of the medical care of that illness or injury.

WHAT ARE MY RIGHTS AS A RESEARCH SUBJECT?
Taking part in this study is completely voluntary. You may choose not to take part in this study or leave this study at any time. You will be treated the same no matter what you decide.
We will tell you about new information from this or other studies that may affect your health, welfare, or willingness to stay in this study. If you would like the results of the study, let the study staff know.

WHAT DO I DO IF I HAVE QUESTIONS OR PROBLEMS?
For questions about this study or a research-related injury, contact:

- Pablo Tebas, MD (215-615-4321)
- Clinical Trials Unit (215 349-8092)

For questions about your rights as a research subject, contact:

- Director of Regulatory Affairs at the University of Pennsylvania by phoning (215) 898-2614

SIGNATURE PAGE FOR STUDY

If you have read this consent form (or had it explained to you), all your questions have been answered and you agree to take part in this study, please sign your name below.

______________________	______________________
Participant's Name (print)	Participant's Signature and Date
______________________	______________________
Participant's Legal Guardian (print)<br>(As appropriate)	Legal Guardian's Signature and Date
______________________	______________________
Study Staff Conducting<br>Consent Discussion (print)	Study Staff Signature and Date

APPENDIX III: DSMB MEMBERSHIP AND CHARTER

DSMB members: **Greg Bisson, MD**

Harvey Friedman, MD

Knashawn H Morales , PhD

DSMB Charter and Charge

Per DSMB charter, the DSMB is respectfully asked to review the study progress to date.

As specified in the protocol, a data monitoring Committee (DSMB) will be constituted at the University of Pennsylvania and will review trial data in an ongoing basis. This Committee will be composed of no less than three members who are not affiliated with the study, one of whom is a biostatistician. This committee will have a mechanism in place to immediately notify the PI site regarding findings relevant to the safety of study participants, and will have criteria to recommend for stopping the study, should that become necessary. The DSMB will be provided reports as it requests and the DSMB will produce an annual report for the IRB. This report will include:

-Protocol title, HSC protocol number, and activation date of the study.
-Number of patients enrolled to date on each cohort
- Date of first and most recent patient enrollment
- Summary of all adverse events regardless of grade and attribution for to each cohort
-Summary of any recent literature that may affect the ethics of the study.

You will be asked to review this report and decide if the study should continue as planned, or a modification is required.

A reminder of the protocol specified stopping rules follows:

"Stopping rules guidelines are based on exact 95% binomial intervals. If the lower bound of the exact 95% binomial interval exceed 20% at any point, either within a treatment cohort or across cohorts combined, the study will be suspended to further accrual pending DSMB review. So, for example if 4 of the first 5 or 6 subjects within a treatment cohort have a serious adverse event requiring discontinuation, the study will be suspended. However, if 4 of the first 7 subjects within a cohort are discontinued, the study will not be suspended, since a toxicity rate of <20% cannot be ruled out with 95% confidence.If the frequency of serious adverse events that warrants treatment discontinuation is greater than 33% (4 subjects) in any given arm, that arm will be closed. The events will include too the development of a new opportunistic infection, such as tuberculosis, a significant drop (greater than 50% of the CD4 + cell count and an increase in the HIV-RNA viral load greater than 1 log. As the investigators will be blinded to the patient treatment assignment this will be done by the DSMB. "

APPENDIX IV: DATA SAFETY MONITORING PLAN

DATA AND SAFETY MONITORING PLAN

A PHASE IB, RANDOMIZED, PLACEBO CONTROLLED, DOUBLE BLIND STUDY TO DETERMINE THE SAFETY, VIRAL SUPPRESSION, PHARMACOKINETICS AND IMMUNE MODULATORY EFFECTS OF TREATMENT WITH APREPITANT (EMEND®) IN HIV INFECTED INDIVIDUALS

IND # 75,558;
Clinical and Translational Research Center Study # 1040 University of Pennsylvania IRB Protocol # 805450

Principal Investigator
Pablo Tebas, M.D.
University of Pennsylvania

Principal Investigator of Program Project
Steven D. Douglas, M.D.
University of Pennsylvania
The Children's Hospital of Philadelphia

Supported by the National Institute of Mental Health
(P01-MH76388 "Neurokinin 1-R antagonists for HIV therapy")

December 10, 2007

Purpose

The monitoring of a clinical trial is an essential element of study processes designed to ensure the protection of the subject's rights, the safety of subjects enrolled in the trial and the integrity and quality of the resulting data. This monitoring plan details the Case Report Form (CRF) and source data verification of efficacy and safety parameters, the frequency of monitoring visits, regulatory document review, drug accountability and compliance review.

Monitoring will be conducted according to the University of Pennsylvania Sponsor-Investigator Standard Operating Procedures.

Protocol Summary

Preliminary studies indicate that NK-1R substance P antagonists have a significant antiviral activity in vitro. This proof of concept proposal is centered on addressing the safety and antiviral activity of using aprepitant, a NK-1R substance P antagonist as an antiviral agent for the treatment of HIV infection. Understanding the pharmacokinetics and pharmacodynamics of aprepitant in HIV infected individuals and its relationship with its potential antiviral effect is a critical part of this project.

In vitro studies suggest that the antiviral activity of SP antagonists might involve chemokine receptor expression. Substance P antagonists decrease the expression of CCR5 in macrophage cells, a mechanism that might explain, at least in part the anti HIV activity of these compounds. Our preliminary data also suggest that SP may have direct effects upon NK cells as determined using a clonal NK cell line. Since we have found that SP antagonism can prevent some of this activity, it is alluring to speculate that SP antagonism may serve to reverse the impairment of NK cell function found in HIV infection. This study will provide a unique opportunity to evaluate the in vivo effects of aprepitant on CCR5 expression and message in human monocytes and lymphocytes and to study NK cell function after the administration of this drug. We will also conduct in the laboratory of Dr. Jordan Orange in vitro experiments to better understand the effects of SP and its antagonism in NK cells as well as the mechanism of these activities.

HIV infection has a significant impact of depression and anxiety disorder symptoms. In women with HIV infection the proportion of major depression is four times higher than in HIV-negative women (19.4 vs 4.8%). This study provides a unique opportunity to explore the effects of NK-1 receptors antagonists on depression, anxiety and sleep in HIV infected subjects.

The objective of this randomized, placebo controlled, double blind study is to determine the antiviral activity of aprepitant by comparing the change in HIV RNA viral load after 2 weeks of aprepitant monotherapy.

27 patients with HIV-1 infection, not receiving antiretroviral therapy and with CD4+ cell count ≥ 350/mm^3 and plasma HIV-1 RNA of ≥2000 copies/mL will be randomized 1:1:1 to receive two different doses of aprepitant (Emend®) or placebo. [Arm A: Placebo; Arm B: Aprepitant 125 mg QD or Arm C: Aprepitant 250 mg QD]

Monitor Selection and Training

One monitor will be assigned for this trial and will be responsible to complete the monitoring process. The monitor is someone who is independent from the trial and the study team. The monitor will be selected from one of the preferred vendors already working with the University of Pennsylvania.

A CV for the monitor will be obtained and updated annually. The CV will be kept on file in the Sponsor section of the Regulatory Binder to document the qualifications of the monitor.

The monitor will receive a monitoring manual, which will include a copy of the protocol, the approved informed consent form, approved CRFs, and this monitoring plan. ***.*** *A training session will be scheduled as soon as is practicable that will include the PI giving an overview of the protocol, and the study statistician providing training on the actual monitoring plan, including reviewing the CRFs with the monitor. It is estimated that the training session will take a half day.*

Study Initiation

The Principal Investigator will be responsible for assuring through personal contact between the co-investigators, the monitor and the clinical staff that each clearly understands and accepts the obligations incurred in the undertaking of this clinical trial.

The Principal Investigator should ensure that the clinical staff fully understand the nature of the protocol and the requirements for an adequate and well-controlled study; the obligation to conduct the clinical investigation in accordance with the applicable federal regulations; the obligation to obtain informed consent in accordance with 21 CFR Part 50; the obligation to obtain IRB review and approval of a clinical investigation before the investigation may be initiated and to ensure continuing review of the study by the IRB in accordance with 21 CFR Part 56.

A check list containing the elements of study initiation as described in the CFR and ICH GCPs will be completed by the study staff prior to the first the monitoring visit and placed in the Sponsor section of the Regulatory Binder.

Monitoring Visits

5.1 FREQUENCY

Enrollment will be complete when 27 subjects are enrolled into the trial. Approximately 1-2 subjects will be enrolled per month. Monitoring visits will be conducted periodically throughout the study as described below. (Note: The specific data to be reviewed at each visit is indicated in Section 5.2)

- *The **first monitoring visit** will occur when one-third of the subjects have been enrolled (n=9).*
- *A **second monitoring visit** will be conducted when approximately two-thirds of the subjects have been enrolled (n=18).*

- *A **third monitoring and closeout visit** will be conducted after 100% of the subjects have been enrolled (n=27). This visit will be conducted after the subjects have completed the study and will also serve as the close-out monitoring visit (as required by GCPs and described in section 10).*

5.2 DATA REVIEW FOR MODERATE RISK STUDY

The following variables will be source data verified:

- *Screening Consent (if applicable)*
- *Informed Consent*
- *Inclusion/Exclusion Criteria*
- *Drug Accountability*
- *Serious and Non-Serious Adverse Events*
- *Stopping rule criteria*
- *Laboratory results*
- *Physical examination and vital signs*
- *Concomitant medications (to ascertain that no prohibited medications given)*
- *End of study form*

5.3 REGULATORY DOCUMENTS REVIEWED

The Regulatory Documents will be maintained in the Regulatory Binder. The Regulatory Binder will be reviewed by the monitor in an ongoing manner at the time of each monitoring visit. The monitor will review the regulatory binder for completeness and will assure that the CRFs are being completed in a timely manner.

5.4 DOCUMENTATION OF THE MONITORING VISIT

Monitoring Log
The monitor is required to sign and date the monitoring log documenting the dates of the monitoring visit. The Monitoring Log will be filed in the Regulatory Binder.

Monitoring Report
All monitoring visits will be documented on the Monitor's Report and Visit Checklist. The original report for each visit will be filed in the Sponsor section of the Regulatory Binder and copies of the report will be sent to:

- *The Office of Human Research at the University of Pennsylvania, School of Medicine.*
- *The overall Program Project, of which this study is a component, Principal Investigator, Steven D. Douglas, MD, at The Children's Hospital of Philadelphia.*

Data Management

Data management is performed by the Biostatistics and Data Management Core (BDMC) of the Joseph Stokes, Jr. Research Institute at The Children's Hospital of Philadelphia. The completed CRFs are brought to the BDMC by the study team and are stored at the BDMC after double data entry.

Mr. John Nevy, manager of the Data Management sub-Core within the BDMC is responsible for the clinical database, data entry, queries and database corrections and for maintaining a data management plan.

Drug Accountability

Study drug for this trial consists of Aprepitant 125 mg and matching placebo which are supplied and available through a licensed pharmacist at the University of Pennsylvania, Deborah Kim. All study drug/placebo are stored in the pharmacy at the Clinical Trials Office at the University of Pennsylvania. The aprepitant and the placebo for aprepitant must be stored under controlled room temperature, 20-25°C (68-77°F) and protected from light, humidity and excessive heat. The pharmacist is required to maintain complete records of all study products.

The monitor will perform 100% drug accountability in an ongoing manner at the time of each monitoring visit. The monitor will confirm the receipt, dispensation, and return of drug. The pharmacist will retain returned drug until the monitor can verify the returns which can then be destroyed. Destruction will be conducted by placing returns in a red biohazard bag, which is then incinerated by Stericycle Company.

Medical Monitoring

Patient safety will be monitored continuously by the Medical Monitor, Ian Frank, MD, who is delegated and qualified to review medical safety data for the duration of the study.

The Principal Investigator has the front-line responsibility for identifying potential adverse events experienced by study participants, adjusting the intervention accordingly and reporting the experience. The Principal Investigator is responsible for tracking these reports and relaying them as required to the FDA, University of Pennsylvania IRB, the Data and Safety Monitoring Board (see Section 9) and the Program Project Principal Investigator, Steven D. Douglas, MD.

Data and Safety Monitoring Board

This study has a Data and Safety Monitoring Board (DSMB) comprised of three members: Greg Bisson, MD (chair), Harvey Friedman, MD, Knashawn H Morales, PhD (statistician). The DSMB is charged with reviewing the trial data in an ongoing basis. If the committee reviews findings relevant to the safety of study participants, it will immediately notify the PI regarding those, and will have criteria to recommend for stopping the study, should that become necessary. The DSMB will be provided reports as it requests and the DSMB will produce an annual report for the IRB. This report to the DSMB will include the number of patients enrolled to date in each stratum, the dates of first and most recent patient enrollment, a summary of all adverse events regardless of grade and attribution for to each cohort, and by treatment, and a summary of any recent literature that may affect the ethics of the study. The summary of adverse events by treatment will be provided by the study statistician directly to the committee and not as part of the report provided to study PI, since this study is a randomized blinded study.

Close Out Visit

The monitor will conduct the Close -Out Visit at the time of the third monitoring visit and within 3 months after the last patient has completed the study.

The following activities will be completed by the monitor to close out the study:

- Ensure that all data has been reviewed and collected;
- Ensure that all outstanding queries are answered;
- Confirm all Serious Adverse Events, MedWatch Reports and IND Safety Reports, if applicable, have been reported to the IRB(s), National Institute of Mental Health, and the FDA;
- Review the Regulatory documentation and Subject Files for completeness and compliance with GCP and all applicable federal regulations;
- Ensure initial and revised 1572 forms were submitted to the IRB;
- Ensure all protocol violations were submitted to the IRB;
- Ensure that all continuing review reports were submitted to the IRB;
- Perform drug accountability and destroy any unused drug.
- Review requirements for record retention with the investigator and the clinical staff.

A copy of the report will be sent to the Office of Human Research as well as to the Principal Investigator of the Program Project of which this study is part, Steven D. Douglas, MD, The Children's Hospital of Philadelphia.

Compliance Monitoring Review

Good Clinical Practice oversight monitoring is conducted for randomly selected studies by the Office of Human Research (such as Informed Consent Monitoring or Monitoring Plan implementation). In addition, studies may be randomly selected and reviewed by other designated offices in the School of Medicine for compliance aspects.

APPENDIX V: QUESTIONNAIRES

1. Hamilton-17 Depression Rating Scale (HAM-D-17) score (7 pages)
2. HAM-A Anxiety Symptoms score (1 page)
3. Pittsburgh Sleep Quality Index score (2 pages)
